# Genomic and transcriptomic profiling of resistant CEM/ADR-5000 and sensitive CCRF-CEM leukaemia cells for unravelling the full complexity of multi-factorial multidrug resistance

**DOI:** 10.1038/srep36754

**Published:** 2016-11-08

**Authors:** Onat Kadioglu, Jingming Cao, Nadezda Kosyakova, Kristin Mrasek, Thomas Liehr, Thomas Efferth

**Affiliations:** 1 Department of Pharmaceutical Biology, Institute of Pharmacy and Biochemistry, Johannes Gutenberg University, Mainz, Germany; 2 Jena University Hospital, Friedrich Schiller University, Institute of Human Genetics, Jena, Germany

## Abstract

We systematically characterised multifactorial multidrug resistance (MDR) in CEM/ADR5000 cells, a doxorubicin-resistant sub-line derived from drug-sensitive, parental CCRF-CEM cells developed *in vitro*. RNA sequencing and network analyses (Ingenuity Pathway Analysis) were performed. Chromosomal aberrations were identified by array-comparative genomic hybridisation (aCGH) and multicolour fluorescence *in situ* hybridisation (mFISH). Fifteen ATP-binding cassette transporters and numerous new genes were overexpressed in CEM/ADR5000 cells. The basic karyotype in CCRF-CEM cells consisted of 47, XX, der(5)t(5;14) (q35.33;q32.3), del(9) (p14.1), +20. CEM/ADR5000 cells acquired additional aberrations, including X-chromosome loss, 4q and 14q deletion, chromosome 7 inversion, balanced and unbalanced two and three way translocations: t(3;10), der(3)t(3;13), der(5)t(18;5;14), t(10;16), der(18)t(7;18), der(18)t(21;18;5), der(21;21;18;5) and der(22)t(9;22). CCRF-CEM consisted of two and CEM/ADR5000 of five major sub-clones, indicating genetic tumor heterogeneity. Loss of 3q27.1 in CEM/ADR5000 caused down-regulation of *ABCC5* and *ABCF3* expression, Xq28 loss down-regulated *ABCD1* expression. *ABCB1*, the most well-known MDR gene, was 448-fold up-regulated due to 7q21.12 amplification. In addition to well-known drug resistance genes, numerous novel genes and genomic aberrations were identified. Transcriptomics and genetics in CEM/AD5000 cells unravelled a range of MDR mechanisms, which is much more complex than estimated thus far. This may have important implications for future treatment strategies.

Leukaemia constitutes a heterogeneous group of haematopoietic malignancies and can be categorised in four main types: acute myeloid leukaemia (AML), acute lymphoblastic leukaemia (ALL), chronic myeloid leukaemia (CML) and chronic lymphocytic leukaemia (CLL)^1^. ALL is referred as the most common paediatric oncological diagnosis^2^,^3^ and overall survival of ALL patients remains relatively poor with 20–40%^4^. In USA, leukaemia is the sixth leading cause of cancer associated death with incidences of 7.1 per 100,000 people per year and one of the main cause of death worldwide among children^5^.

Drugs accumulate in cancer cells by various mechanisms, such as diffusion, transport and endocytosis. Each of these mechanisms possesses physiological significance based on detailed uptake studies in drug-resistant mutants^6^. Main reasons of chemotherapy failure are drug resistance of tumour cells and the high susceptibility of normal tissues to treatment-related toxicity^7^,^8^,^9^. Important multidrug resistance mechanisms in cancer are apoptosis inhibition, DNA repair, drug efflux, altered drug metabolism and others^6^,^10^. Some immunotoxin-based anti-cancer drugs enter cells by receptor mediated endocytosis to kill tumour cells^11^. Vesicle trafficking, including the release of extracellular micro-vesicles, is critical in carcinogenesis, which involves invasion, metastasis, cell cycle regulation, angiogenesis, tumour immune privilege, neoplastic coagulopathy and multidrug resistance (MDR)^12^. Moreover, one study in eukaryotic cells pointed out that the balance between exocytosis and endocytosis is critical for generating the membrane domains recognized by sterol-targeting antibiotics, determining their efficacy^13^. Therefore, regulation of endocytosis and exocytosis may be considered as another mechanism of drug resistance.

In order to maximise the therapeutic benefit and minimise treatment-related toxicity, drug resistance phenomena should be better understood and the responsible mechanisms should be identified. For this purpose, gene expression profiling of different kinds of tumours needs to be investigated to unravel the multi-facetted nature of drug resistance in a more comprising manner.

Molecular cytogenetic studies provide an important approach to characterise drug resistance of tumours^14^. MDR is primarily mediated by P-glycoprotein, which acts as energy-dependent efflux pump to reduce intracellular drug concentrations^15^,^16^,^17^. In addition, random chromosomal rearrangements leading to capture and activation of *ABCB1*/*MDR1* gene have been proposed as mechanism of MDR^18^.

RNA sequencing represents a powerful and sensitive method for gene expression profiling^19^,^20^,^21^. It has been used in combination with cytogenetic profiling to evaluate differential gene expression profiles and chromosomal aberrations in leukaemia cells^22^,^23^,^24^,^25^,^26^. Array-comparative genomic hybridisation (aCGH) and multicolour fluorescence *in situ* hybridisation (mFISH) techniques are valuable to detect genetic aberrations associated with the acquisition of drug resistance^27^,^28^. Such genetic aberrations provide clues about putative drug resistance genes in affected chromosomal regions. However, there is scarce information on the systematic analysis of MDR cells by parallel assessment of transcriptome-wide RNA sequencing and cytogenetic profiling by aCGH and mFISH.

While it is known that drug resistance can be multifactorial in nature, the full complexity of mechanisms and genetic alterations have been rarely addressed as of yet. In this study, we applied RNA sequencing, aCGH and mFISH to analyse drug sensitive parental CCRF-CEM and multidrug-resistant CEM/ADR5000 cells.

## Results

### Differential gene expression profile of CEM/ADR5000 cell line and downstream pathway analysis

**Figure 1:**
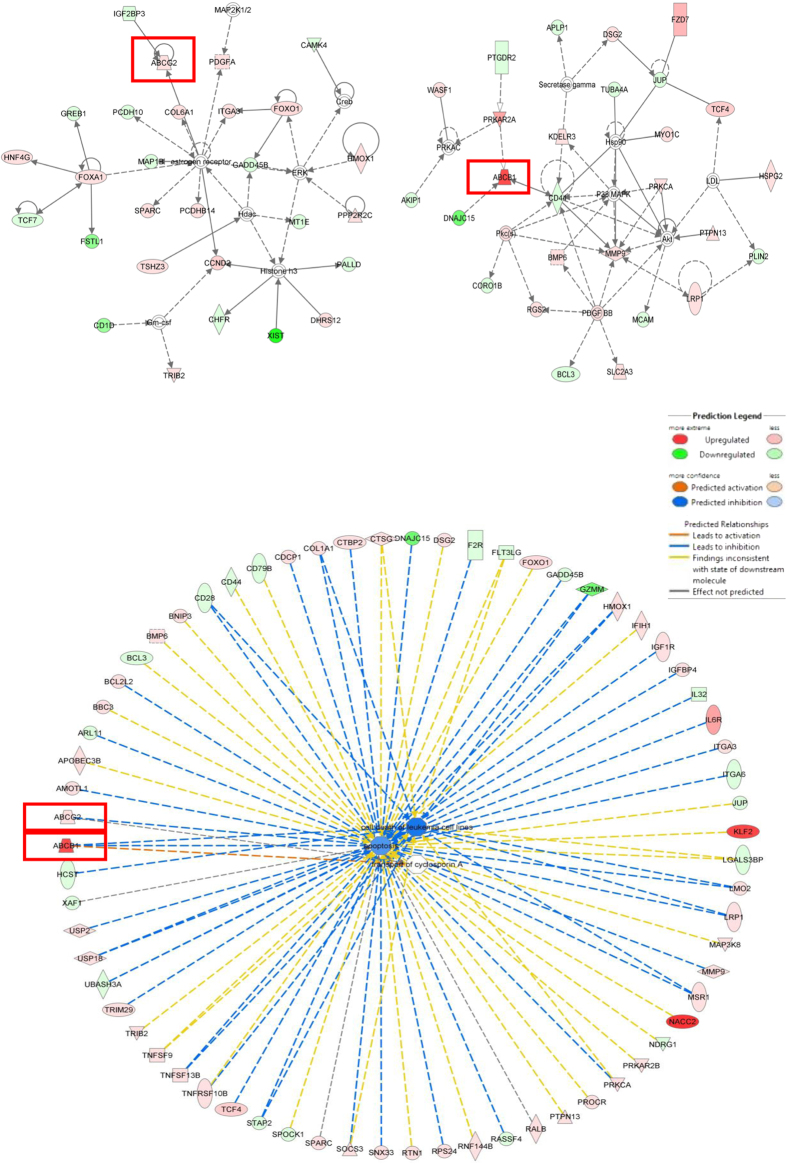
Gene networks influenced by *ABCB1* and *ABCG2* in CEM/ADR5000 cells. IPA software was used to depict the networks. Genes that are labelled in green were down-regulated and genes that are labelled in red were up-regulated. The lower panel depicts *ABCB1* and *ABCG2* playing role in “cell death of leukaemia cell lines” and “apoptosis” inhibition as shown by blue dotted lines. *ABCB1* up-regulation is predicted to activate “transport of cyclosporine A” as shown by the orange dotted line.

The RNA sequencing data were analysed by considering RPKM (reads per kilobase of exon model per million mapped reads) values. Ratios of overall RPKM values for the expression of each gene in CEM/ADR5000 cells in comparison to that of CCRF-CEM were considered as fold change of gene expression. Firstly, setting a fold change threshold of ±1.5 yielded in 3,186 differentially expressed genes in CEM/ADR5000 cells. A threshold of ±3 resulted in 1,199 and a threshold of ±7 in 509 deregulated genes. Finally, if a fold change threshold of ±10 was applied, 369 deregulated genes were recorded. For further analysis, only the ±7 threshold was taken into account. Deregulated gene lists were used for downstream pathway analysis with Ingenuity Pathway Analysis (IPA) to identify affected pathways and networks in CEM/ADR5000 cells, if compared to CCRF-CEM cells. Downstream pathway and network analyses yielded similar results for ±7 and ±10 fold changes. Here, we show only the results for the ±7 fold change threshold. Three ATP-binding cassette (ABC) transporters (*ABCA2, ABCB1* and *ABCG2*) were among the most up-regulated genes. They were 10.5-, 402.4-fold and 12.2-fold up-regulated, respectively, in CEM/ADR5000 cells in comparison to CCRF-CEM cells. Pathway and network analyses of deregulated genes in CEM/ADR5000 cells revealed connections to drug resistance and carcinogenesis, *e.g.* “cell death of leukaemia” and “apoptosis” pathways were inhibited, whereas the “transport of cyclosporine” network was predicted to be activated due to up-regulated *ABCB1*. The networks involving *ABCB1* and *ABCG2* are summarised in Fig. 1.

Several genes known to be involved in drug resistance were deregulated implying that CEM/ADR5000 cells exerts a multi-factorial resistance phenotype. If a fold change threshold of ±7.0 was applied, 7 out of 101 apoptosis-regulating genes (7%), 34 out of 726 kinases (5%) and 3 out of 48 ABC transporters (6%) were deregulated implying that genes from these gene classes may have an important influence on the MDR phenotype of CEM/ADR5000 cells. These genes are depicted in Table 1. A full list of all deregulated genes involved in resistance mechanisms is given in Supplementary Table 1.

**Figure 2:**
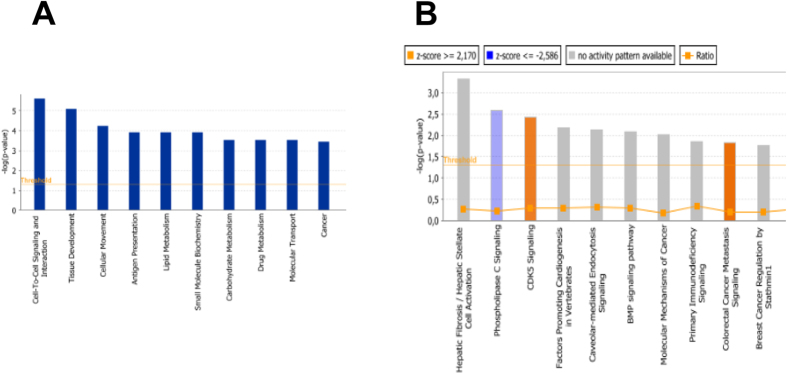
(**A**) Biological function of differentially expressed genes in CEM/ADR5000 cells in comparison to wild-type CCRF-CEM cells as determined by IPA software. The orange line depicts the statistical significance threshold (p = 0.05). **(B)** Signaling pathways of differentially expressed genes in CEM/ADR5000 cells in comparison to wild-type CCRF-CEM cells as determined by IPA software. The orange line depicts the statistical significance threshold (p = 0.05) and the orange chart depicts the ratio of deregulated genes in each pathway.

Lipid metabolism, small molecule biochemistry, carbohydrate metabolism, drug metabolism, molecular transport, cancer, haematological disease, cellular development, cellular growth and proliferation, cell death and survival were identified by IPA as biological functions that involve *ABCB1*. A bar chart for the most affected biological functions and pathways is depicted in Fig. 2A,B.

Three genes involved in DNA repair were up-regulated in CEM/ADR5000 cells, which emphasises the role of DNA repair as important mechanism of drug resistance: *NEIL2* was up-regulated by 22.35-fold, *TEX15* by 10.52-fold.

Genes playing a role in membrane lipid metabolism *via* the ceramide pathway were down-regulated in CEM/ADR5000 cells. *SMPD3* was down-regulated by 5.71-fold and *ACER1* by 3.17-fold.

*NQO1*, which plays role in reactive oxygen species pathway and apoptosis regulation, is down-regulated by 3.57-fold in CEM/ADR5000 cells.

Functional enrichment analyses using the DAVID software pointed to various resistance related biological functions. “Leukocyte differentiation” (p = 7.4 × 10^−5^; fold-enrichment: 3.8), “regulation of exocytosis” (p = 2.3 × 10^−3^; fold-enrichment: 6.3), and “membrane organisation” (p = 2.4 × 10^−3^; fold-enrichment: 2.1). The results are summarised in Table 2.

The analysis of the drug resistance gene list of SABioscience (http://www.sabiosciences.com/ArrayList.php) revealed 9 down-regulated and 25 up-regulated genes, if fold change thresholds of ±7 were applied. The results are shown in Table 3. *DNAJC15* (down-regulated by 499-fold), *ABCB1* (up-regulated by 402-fold), *PDLIM1* (upregulated by 270-fold), FZD7 (up-regulated by 161-fold) and *CCND2* (up-regulated by 101-fold) were the most deregulated genes residing at drug resistance clusters.

**Figure 3:**
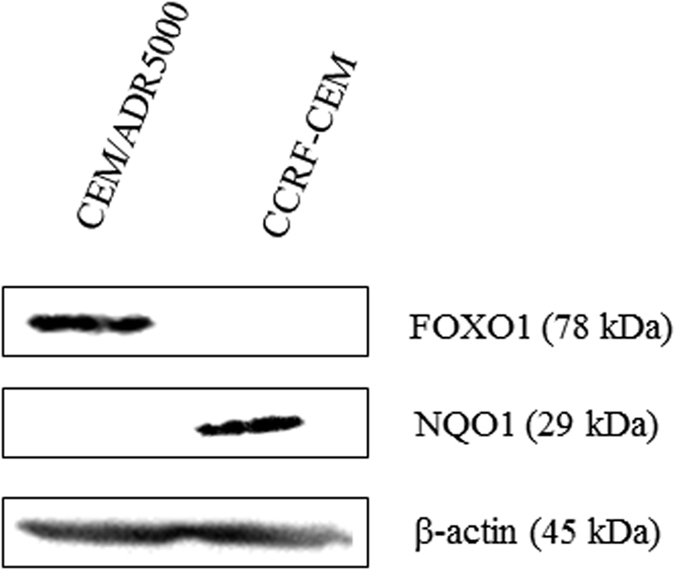
Protein expression of FOXO1 and NQO1 in CEM/ADR5000 and CCRF-CEM cells as determined by western blotting (cropped blots are displayed).

Validation of the selected resistance genes were performed at the protein level for FOXO1 and NQO1. As shown in Fig. 3, FOXO1 was up-regulated, whereas NQO1 was down-regulated in CEM/ADR5000 cells, correlating with the RNA sequencing output and validating the RNA expression data at the protein level.

### mFISH

CCRF-CEM cells revealed the following karyotype by mFISH: 47, XX, der(5)t(5;14) (q35.33;q32.3), t(8;9) (p12;p24), del(9) (p14.1), +20[85%]/46, X, -X, der(5)t(5;14) (q35.1;q32.3), del(9) (p14.1), +20[15%]. A deletion in the chromosomal region 9p and chromosome 20 trisomy were also confirmed by aCGH analysis.

CEM/ADR5000 cells showed a less stable profile with the following highly complex karyotype in clone 1, which represents about 19% of the cells; 47, X, -X, t(3;10) (q11.2 ~ 12;p14 ~ 15), der(3)t(3;13) (q26.32;q22.3), del(4) (q31.32q34.3), der(5)t(18;5;14) (18qter→18q21.2::5p12→5q35.33::14q32.3→14qter), inv(7) (p21.1q21.1), t(8;9) (p12;p24), del(9) (p14.1), t(10;16) (q23.31;q22 ~ 23), del(14) (q32.3), der(18)t(7;18) (p21;q21.2), der(18) (21qter→21q22.1::18p11.22→18q12.1::5p12→5pter), der(18) (21p?::21q22.3→21q22.1::18p11.22→18q12.1::5p12→5pter), +20, der(22)t(9;22) (q22.33;q13.33). Besides, there were four additional clones with the following genetic aberrations compared to clone 1:

- Clone 1a (20%) with an additional translocation t(6;14) (q26;q32.33);
- Clone 1b (26%) with a translocation between one chromosome 20 and a derivative chromosome der(10)t(3;10);
- Clone 1b1 (30%) with the same additional aberration as clone 1b and an additional translocation between a chromosome 17 and der(18) (21;18;5);
- Clone 1c (5%) with a translocation t(6;20;8) (q24;q11.2 ~ 1;q22.3 ~ 23) and loss of the derivative chromosome der(18) (21qter→21q22.1::18p11.22→18q12.1::5p12→5pter).

**Figure 4:**
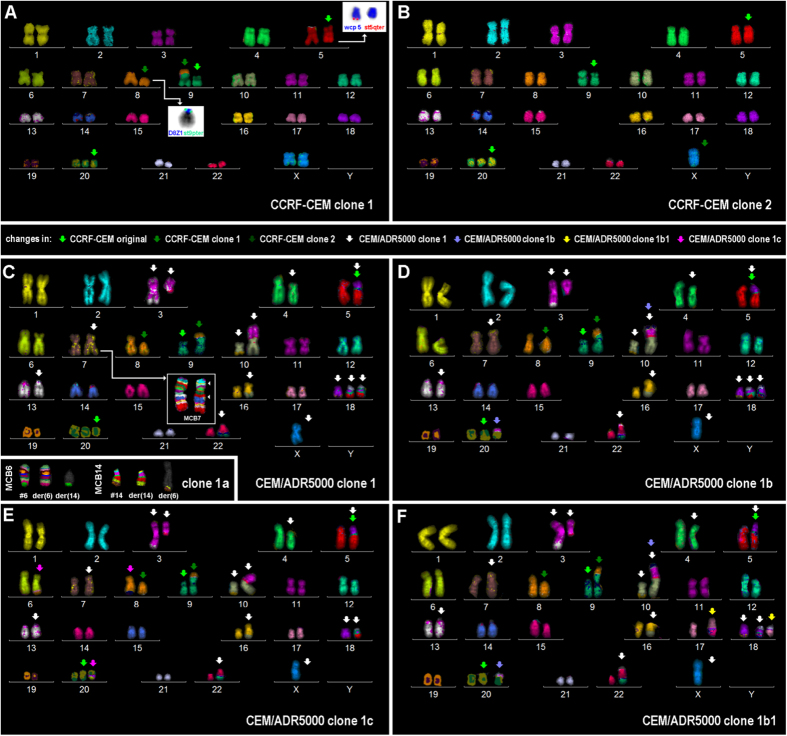
mFISH analysis of CCRF-CEM and CEM/ADR5000 cells. Two clones detected in CCRF-CEM are depicted in (**A**,**B**). All derivative chromosomes present in clones 1 and 2 are highlighted by light-green arrows. Individual changes for clones 1 and 2 are labelled by arrows in darker green. For derivative chromosome 5, a whole chromosome paint (wcp) and a subtelomeric (st) probe for 5qter were applied. For the derivative chromosome 8, a centromeric probe (D8Z1) and a st probe for 14qter had been used. In (4C) to F, CEM/ADR5000 clones 1, 1a, 1b, 1b1 and 1c are depicted. The per clone acquired alterations are highlighted by coloured arrows as explained in the legend between (4A/B) and C/D. For clear visualisation of the inversion in chromosome 7, MCB 7 was applied as shown in (4C). In (4C), the only additional aberration present in clone 1a is depicted, *i.e.* a reciprocal translocation between chromosomes 6 and 14.

Deletion at chromosomal regions 3q and 9p, deletion and amplifications in chromosome 18, chromosome 20 trisomy and loss of one X chromosome were confirmed by aCGH analysis. The results for the mFISH analyses are summarised in Fig. 4.

**Figure 5:**
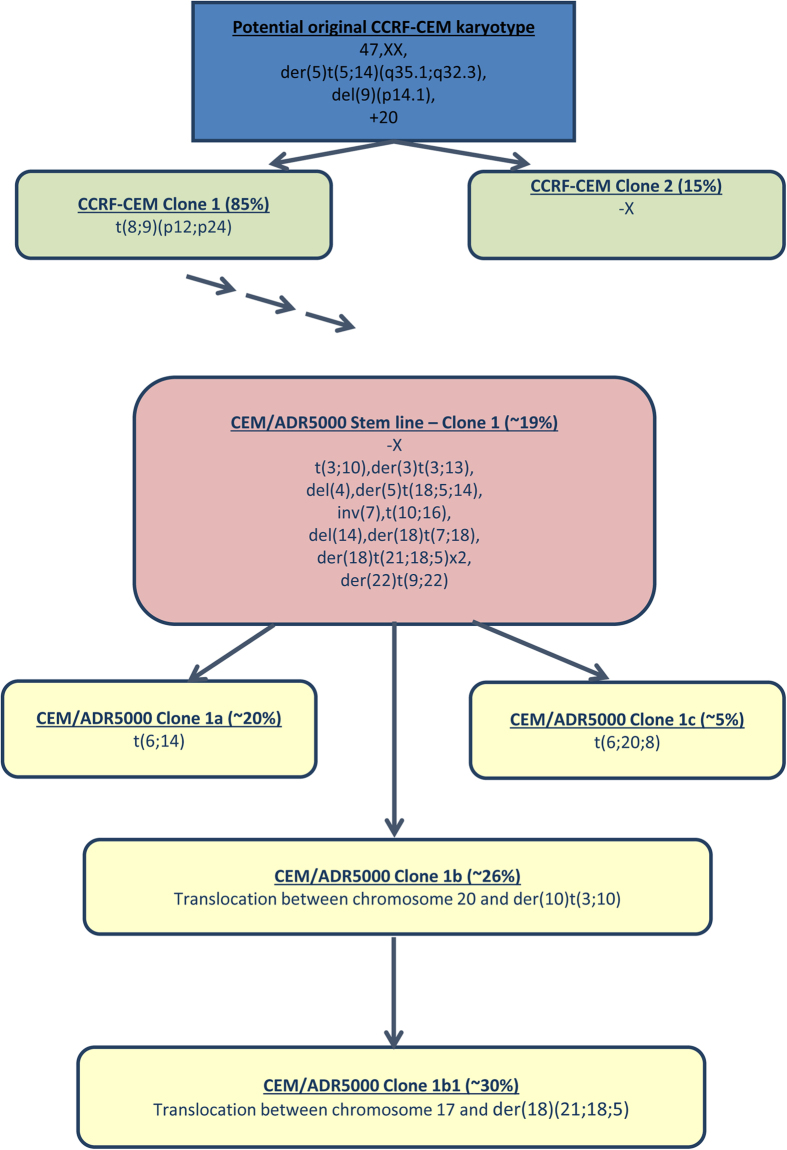
Summary of clonal evaluation of cell lines CCRF-CEM and CEM/ADR5000.

The clonal evolution of CCRF-CEM and CEM/ADR5000 cells are summarised in Fig. 5 and detailed karyotypes of all subclones detected in this study are listed in Supplementary Table 2.

### aCGH of CCRF-CEM cells

**Figure 6:**
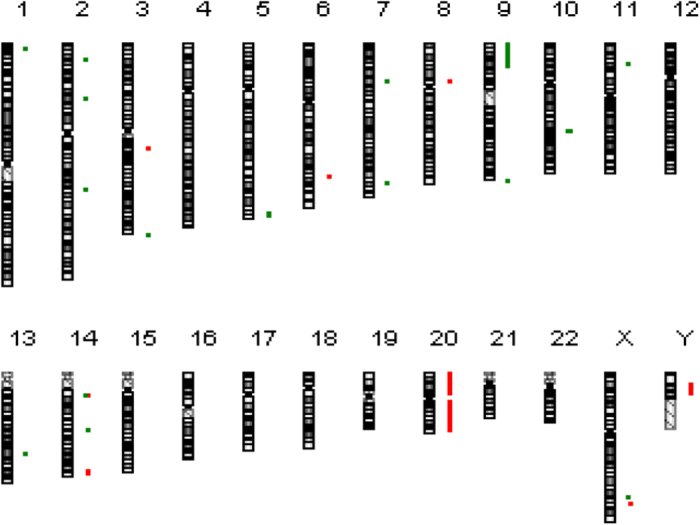
aCGH results of CCRF-CEM cells.

One deletion was located between 9p21.1 and 9p24.3 (28,466,044 bp) with 21 deregulated genes, 12 of which were down-regulated as shown by RNA sequencing. An amplification was detected between 20p11.1 and 20p13 (26,126,681 bp) carrying 22 deregulated genes. Of them, 11 were found by RNA sequencing to be up-regulated, including *CD93* as highest up-regulated gene (29.9-fold). Another amplification was located between 20q11.21 and 20q13.33 (33,061,715 bp). This region harboured 67 deregulated genes, and 45 of them were found by RNA sequencing to be up-regulated. This amplification contained the *MYH7B* and *C20orf197* genes with 10.8 and 9.7 fold upregulation, respectively. The results are summarised in Fig. 6 and Table 4. Deletions are represented in green and amplifications in red.

### aCGH of CEM/ADR5000 cells

CEM/ADR5000 cells possessed considerably more deletions and amplifications than CCRF-CEM cells, indicating high selection pressure during resistance development. The corresponding chromosomal locations with amplifications and deletions were compared with those of the deregulated genes identified by RNA-sequencing. The aCGH results were corroborated by RNA sequencing results, since most deregulated genes were located within the chromosomal loci, which were identified to be amplified or deleted by aCGH. One deletion was detected between 1p36.31 and 1p36.32 (2,005,754 bp), and this region harboured six deregulated genes. Five of them were found by RNA sequencing to be down-regulated. Another deletion was detected between 3q26.32 and 3q29 (21,664,432 bp), and this region carried 72 deregulated genes. Of them, 68 were found by RNA sequencing to be down-regulated. A deletion within 3q27.1 caused down-regulation of *ABCC5* and *ABCF3* expression. ABCC5 mediates the ATP-dependent transport of various anticancer drugs, including doxorubicin^29^. Its expression in doxorubicin-resistant human lung cancer cells SBC-3/ADM, AdR MCF-7 and K562/ADM was higher compared to their respective parental cell lines^30^. Since *ABCF3* resides at the same cyto-band with *ABCC5*, their expression might be regulated in a similar manner. However, ABCF3 is not known as MDR related drug transporter. Therefore, the ABCF3 linkage with doxorubicin resistance should not be over-interpreted.

**Figure 7:**
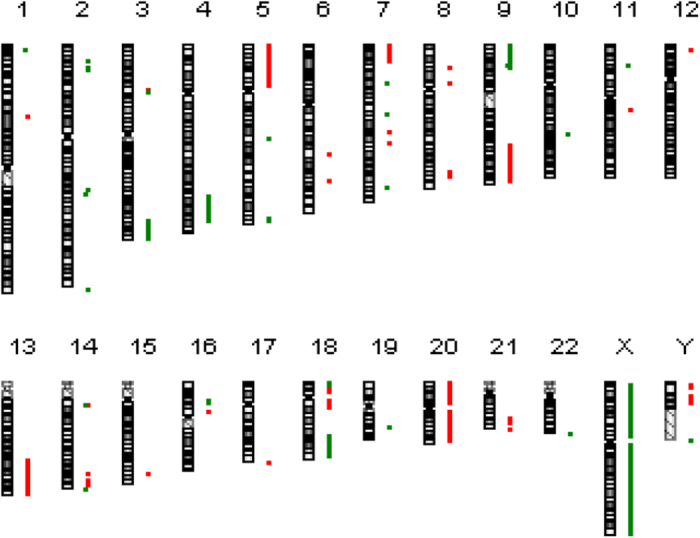
aCGH results of CEM/ADR5000 cells.

Another deletion was detected between 4q31.23 and 4q34.3 (29,086,190 bp). This region harboured 33 deregulated genes, 30 of them were found by RNA sequencing to be down-regulated. One amplification was detected between 7p21.1 and 7p22.3 (16,468,962 bp). This region contained 36 deregulated genes, 31 of them were found by RNA sequencing to be up-regulated. Another amplification was located at 7q21.12 (182,792 bp). This region carried two deregulated genes with *ABCB1* as the most up-regulated gene (402.4-fold). One deletion was found between 16p12.1 and 16p12.3 (6,639,549 bp) and this region involved 25 deregulated genes, 23 of which were found by RNA sequencing to be down-regulated. One deletion was detected between 18p11.22 and 18p11.32 (8,506,661 bp) and this region harboured 16 deregulated genes. All of them were found by RNA sequencing to be down-regulated. Deletion within Xq28 caused down-regulation of *ABCD1* expression. The results are summarised in Fig. 7 and Table 4. Deleted regions are represented in green and amplified regions in red.

A complete list of deregulated genes in ADR/CEM5000 cells in comparison to CCRF-CEM cells is depicted in Supplementary Table 3. The corresponding chromosomal aberrations found by aCGH analyses are depicted in Supplementary Table 4.

### Tumor evolution

Previously, we have already investigated the genetic aberrations of CCRF-CEM and CEM/ADR5000 cells by CGH and mFISH^31^,^32^,^33^. The intention of the present study was to directly compare RNA sequencing data with aCGH and mFISH results from cells harvested at the same time. Nevertheless, we were interested to compare the previous results published in the year 2002 with those of the present investigation. We took this as an opportunity to investigate the evolution of tumor cells over a time period of 14 years permanent culturing *in vitro*.

**Figure 8:**
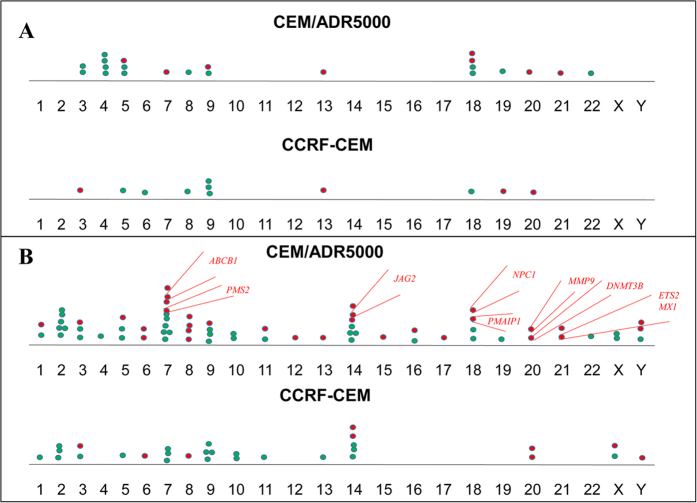
Comparison of chromosomal aberrations analysed in CCRF-CEM and CEM/ADR5000 cells in the year 2002 (**A**) with the results of the present study (**B**). Each dot represents an aberration, green: deletion, red: amplification. Some of the deregulated drug resistance linked genes are marked on the plots for the CEM/ADR5000 cells observed in the present study.

The present study is more detailed and differs from the previously performed CGH and mFISH analysis by us^31^,^32^,^33^ in terms of aberrations. As shown in Fig. 8, the number of chromosomal aberrations both in CCRF-CEM and CEM/ADR5000 increased compared to those studies performed in 2002. Figure 8A depicts the chromosomal aberrations found in the 2002 studies. Figure 8B depicts the chromosomal aberrations found in the present study. One possible explanation is genetic instability that leads to the tumour evolution phenomenon^34^,^35^. Our comparison revealed a considerable number of additional aberrations, which have been acquired over a time period of 14 years of permanent culturing *in vitro*. In the previous studies, we did not observe an aberration in 7q21, which is the region where the *ABCB1* gene resides. In the present study, we could observe an amplification at this region. Chromosomes 7, 14 and 18 involved the majority of the aberrations, chromosome 14 carrying similar number of aberrations for CCRF-CEM and CEM/ADR5000 (three deletions and two amplifications at the former, four deletions and three amplifications at the latter). It can be hypothesised that chromosome 14 might be more prone to genetic instability. Aberrations at this chromosome might be relevant to resist the selection pressure to grow *in vitro*. It warrants more investigations in the future to explore, whether genes at these aberrant chromosomal loci are associated with tumor progression in patients.

## Discussion

Leukaemia is among the most frequent tumours worldwide and the survival rates are still low. One reason is the development of drug resistance towards chemotherapy^36^. P-glycoprotein/*ABCB1/MDR1* is an important determinant of MDR^37^,^38^,^39^. Previous functional studies regarding P-glycoprotein performed by us revealed that natural products targeting P-glycoprotein may serve as good candidate to reverse doxorubicin resistance in CEM/ADR5000 cells^40^. While there is a plethora of reports on single resistance mechanisms, studies focusing on the full complexity of all genetic changes in MDR cells and providing a comprising picture of potential drug resistance mechanisms are still rare.

Gene expression profiling and chromosomal aberration analyses are valuable strategies to identify the genome-wide characteristics of cancer cells. For this purpose, we performed gene expression profiling of multidrug-resistant CEM/ADR5000 and sensitive parental CCRF-CEM leukaemia cells by RNA sequencing and cytogenetic analyses by aCGH and mFISH. Remarkably, CEM/ADR5000 cells showed a less stable chromosomal karyotype with many additional chromosomal aberrations compared to CCRF-CEM cells. Deficiency in *MLH1* and *MSH2* mismatch repair genes might cause a high background mutation rate in CCRF-CEM cells^41^. This deficiency leads to telomere shortening as also observed in human fibroblasts^42^. The background mutation rate was further increased in CEM/ADR5000 cells under drug selection pressure. This led to an increased number of chromosomal aberrations in CEM/ADR5000 cells.

We compared our results with our previous studies in terms of chromosomal aberrations and observed that over a time period of 14 years, those cell lines accumulated additional aberrations. Increased numbers of aberrations on both cell lines might be due to tumour evolution and clonal diversity. One study with acute myeloid leukaemia cells also reported the accumulation of additional aberrations over time^34^. Pre-existing drug-resistant subclones might be a substantial contributor to therapeutic resistance in oncology^43^. It has been stated in the literature that sampling the tumour at different time-points might reveal genetic evolution or differences in the clonal composition of the tumour as the disease progresses^44^,^45^.

*ABCB1*/*MDR1* was the most up-regulated gene in CEM/ADR5000 cells. It was also mapped to pathways and networks linked with MDR and cancer progression, *e.g.* “cell death of leukaemia cell lines”, “apoptosis” and “transport of cyclosporine”. Cyclosporine represents a well-known inhibitor of P-glycoprotein/*ABCB1*^46^,^47^ and up-regulated P-glycoprotein/ABCB1 expression leads to increased cyclosporine efflux^48^. *ABCB1* influenced a number of other biological functions and pathways, and PXR/RXR activation was among these pathways. This pathway is linked with the transport of xenobiotics and endogenous organic compounds^49^,^50^. Downstream pathway and network analyses clearly demonstrated that several drug resistance and cancer progression events were affected in CEM/ADR5000 cells. “Leukocyte differentiation” may be attributed to leukaemia progression, whereas “regulation of exocytosis” and “membrane organisation” may be associated with drug transport.

Several deregulated genes in CEM/ADR5000 are known to be involved in classical resistance mechanisms. DNA topoisomerases, apoptosis-regulating genes, kinases, ABC transporters and autophagy regulating genes were the most frequently influenced resistance mechanisms. Down-regulated DNA topoisomerase 2 has been linked with doxorubicin resistance^51^. Indeed, DNA topoisomerases 2A (*TOP2A*) and 2B (*TOP2B*) were 6.8- and 2.3-fold down-regulated in CEM/ADR5000 cells, respectively. Another study reported that doxorubicin resistant MCF-7 cells showed *ABCB1* (24.0-fold), *ABCG2* (15.8-fold), *RXRA* (4.4-fold), *IGF1R* (3.8-fold) up-regulation and *TP53* (5.9-fold), *MYC* (5.3-fold), *GSK3A* (3.2-fold) down-regulation^52^. This is accordance with our results, since we found up-regulation of *ABCB1* (402.4-fold), *ABCG2* (12.2-fold), *RXRA* (4.4-fold), *IGF1R* (14.6 fold) and down-regulated *TP53* (1.6-fold), *MYC* (1.9-fold), *GSK3A* (2.4-fold) in CEM/ADR5000 cells compared to CCRF-CEM cells.

Glutathione related enzymes represent another class of MDR mediating molecules^53^. Ten glutathione related genes were also deregulated in CEM/ADR5000 cells. Furthermore, apoptotic pathways caused drug resistance in doxorubicin resistant 8226/Dox6 myeloma cells^54^. In concordance with these findings, we observed that the pro-apoptotic genes encoding and caspase 8 were down-regulated by 2.5- and 1.6-fold, respectively, whereas the anti-apototic Bcl2L2 was up-regulated by 24.9-fold.

*HMOX-1/HO-1* (71.7 fold up-regulated in CEM/ADR5000 cells) has been linked with doxorubicin resistance. HMOX-1 may be involved in drug resistance of breast cancer cells by preventing apoptosis and autophagy, since siRNA knockdown of *HMOX-1* enhanced the cytotoxicity of doxorubicin in MDA-MB-231 and BT549 cells^55^,^56^. *HMOX1* possesses anti-apoptotic activity in imatinib-resistant CML patients^57^. Inducing its expression *via* the PKC-β/p38-MAPK (mitogen activated protein kinase) pathway may promote resistance of tumour cells to oxidative stress^57^.

*NQO1* (3.57-fold down-regulated in CEM/ADR5000 cells) plays role in reactive oxygen species pathway and apoptosis regulation. There are various studies pointing out its association with cancer. One study stated that overexpression and genomic gain of *NQO1* locus modulated breast cancer cell sensitivity to quinones^58^. NQO1 protects cells from oxidative stress through inhibition of quinones from entering the one electron reduction to semiquinone free radicals and ROS (reactive oxygen species), therefore NQO1 is considered as an anticancer enzyme^59^,^60^. The use of dietary compounds to induce NQO1 expression has emerged as a promising strategy for cancer prevention by increasing efficacy of bioreductive anticancer drugs^61^,^62^.

*NKX3-1* (2848.9-fold up-regulated in CEM/ADR5000 cells) transcriptionally regulates oxidative damage response and enhances topoisomerase I re-ligation. DNA damage induced by doxorubicin was negatively influenced by *NKX3-1* expression^63^. Another study pointed out that *IGF1R* (14.6-up-regulated in CEM/ADR5000 cells) enhanced the cytotoxicity of doxorubicin in both sensitive and resistant osteosarcoma cells^64^.

*PRKCA* is another gene associated with drug resistance in ovarian cancer cells^65^,^66^, colon cancer cells^67^, and pancreatic cancer cells^68^. Up-regulation in CEM/ADR5000 cells indicates that this gene also contributes to the MDR phenotype of leukaemia cells. *PRKCA* (70.9-fold up-regulated in CEM/ADR5000 cells) phosphorylates and modulates the activity of a doxorubicin transporter, RLIP76. Inhibition of PRKCA and RLIP76 resulted in a synergistic increase of doxorubicin sensitivity^69^.

*TRPV6* has been reported as pro-apoptotic gene in small cell lung cancer cells treated with capsaicin^70^. Its 7.13-fold down-regulation in CEM/ADR5000 cells indicates that it may also contribute to the MDR phenotype of leukaemia.

In total, 37 apoptosis-regulating genes (37% of all apoptosis-regulating genes) were deregulated. Genes involved in other modes of cell death such as autophagy, ferroptosis and necrosis may also contribute to MDR. Twenty-nine percent of autophagy regulating genes were deregulated in CEM/ADR5000 cells, whereas 17% of necrosis or ferroptosis regulating genes were deregulated. This implies that multiple cell death mechanisms may contribute to the MDR phenotype of CEM/ADR5000 cells. Kinases comprise another class of proteins related to drug resistance^71^,^72^. A total of 242 kinases (33% of all kinases) were deregulated, implying a role for MDR of CEM/ADR5000 cells.

Using the SABioscience PCR array list of drug resistance genes (http://www.sabiosciences.com/ArrayList.php) we showed that 9 genes were down-regulated and 25 up-regulated in CEM/ADR5000 cells as observed by RNA sequencing. *PDLIM1* was 270.42-fold up-regulated in CEM/ADR5000 cells. This gene promotes carcinogenesis^73^. *CYP27B1*, which was 13.23-fold up-regulated in CEM/ADR5000 cells, is important for drug metabolism^74^. *BNIP3* plays role in oxidative stress^75^ and was 10.41-fold up-regulated in our resistant cell line. *TCF7* is an anti-apoptotic gene^76^ and was 17.66-fold down-regulated. *SLC2A3* plays a role in carcinogenesis^77^ and was 13.64-fold up-regulated in CEM/ADR5000 cells.

DNA repair was also associated with the MDR phenotype of CEM/ADR5000 cells. Three DNA repair genes were up-regulated, *i.e. NEIL2*^78^,^79^ by 22.35-fold, *TEX15*^80^ by 10.52-fold. Membrane lipid metabolism also contributes to drug resistance^81^,^82^. Two genes playing a role in this metabolic pathway revealed differential expression in CEM/ADR5000 cells. Ceramide is a lipid second messenger, which is synthesised *de novo* or generated from the hydrolysis of sphingomyelin by sphingomyelinases. Ceramide triggers signal transduction pathways in response to cytokines or extrinsic cellular stresses, leading to a variety of cellular responses, including growth suppression and apoptosis^83^. *SMPD3* plays role in ceramide pathway. It is down-regulated and mutated in many leukaemia cells^84^. It was down-regulated by 5.71-fold in CEM/ADR5000 cells, implying a possible role of the ceramide pathway for the MDR phenotype of CEM/ADR5000 cells. Another down-regulated gene of the ceramide pathway was *ACER1* (3.17-fold). It has anti-proliferating and pro-differentiating functions by controlling the generation of sphingosine and/or sphingosine-1-phosphate^85^. Down-regulation of *ACER1* implies that CEM/ADR5000 cells may resist the anti-proliferative activity of *ACER1* better than CCRF-CEM cells.

By using mFISH, we identified novel chromosomal breakpoints in CEM/ADR5000 cells that were not present in parental CCRF-CEM cells, *e.g*. 3q11.2 ~ 12, 3q26.32, 4q31.32, 4q34.3, 5p12, 6q26, 7p21.1, 7q21.1, 9q22.33, 10p14 ~ 15, 10q23.31, 13q22.3, 14q32.33, 16q22 ~ 23, 17q22, 18p11.22, 18q12.1, 18q21.2, 20p11.2, 21q22.1, 21q22.3, and 22q13.33.

4q31-32 has been described as chromosomal locus of a tumour suppressor gene in renal cell carcinoma^86^. The breakpoint at that region may cause loss of this tumour suppressor gene. Amplification of 6q25-27 has been previously associated with platinum resistance in ovarian carcinoma cell lines^87^. Amplification of 7q21.1 as well as chromosomal breaks at this region cause the over-expression of *MDR1/ABCB1*. Over-expression of the *MDR1* gene product, P-glycoprotein, has been associated with abnormalities of chromosome 7, *e.g.* monosomy 7 or 7q8 and 7q9 deletions^88^. Deletion of 10p14-15 has been described in prostate cancer, gliomas and cervical cancer^87^,^89^,^90^. The 10p14-15 break in CEM/ADR5000 cells indicates that this aberration may be critical for leukaemia as well. *PTEN* deletions identified in human prostate cancer suggest unusual genomic features in 10q23.31, which facilitates DNA rearrangements^91^. 14q32.33 amplifications have been observed in platinum-resistant patients, and these amplifications might be a predictive marker of treatment outcome^92^. Loss of 16q was previously correlated with good prognosis in retinoblastoma^93^, supporting the hypothesis that there genes located at 16q22 ~ 23 may be related to cisplatin resistance^94^. 16q22.2 has been linked with increased frequency of loss in drug-resistant serous ovarian carcinoma compared to sensitive ones^95^. The identification of these chromosomal aberrations in our analyses indicates that they are not only relevant for leukaemia too, but also that they may contribute to the MDR phenotype.

By means of aCGH, karyotypic evolution of the cell lines could be detected. The karyotypic subclones of the two cell lines were detected. These subclones were the result of tumour evolution leading to tumour heterogeneity. Genetic alterations in cellular subpopulations allow tumours to better adapt to external selection pressure such as hypoxia, nutritional starvation, and also chemotherapy. Even if chemotherapy would be able to kill the vast majority of tumour cells, few surviving cells of a genetically distinct subclone may repopulate the entire tumour, which ultimately may lead to treatment failure and death of the patient. Therefore, tumour heterogeneity has to be understood as important genetically regulated phenomenon of drug resistance. Our analysis demonstrated that tumour cell heterogeneity is also associated with the MDR phenotype.

Analysing the observed breakpoints, candidate genes may be identified involved in drug resistance, even though point mutations also might play a role–an aspect which cannot be addressed by aGCH and mFISH technologies and which was, therefore, not considered in this study.

In conclusion, the genotypes and gene expression profiles of CCRF-CEM and CEM/ADR5000 leukaemia cell lines were analysed by RNA sequencing, aCGH, and mFISH in a comparative manner. CEM/ADR5000 cells possess an MDR phenotype, which reaches a degree of complexity far higher than estimated in the past for other MDR cell lines. Numerous chromosomal aberrations, deletions, and amplifications associated with the MDR phenotype have been detected. By using RNA sequencing, it was possible to detect gene expression changes with non-precedent preciseness. In addition to known classical drug resistance genes, many new genes were found to be differentially regulated in MDR cells. This study is of high importance for the future development of more specific and promising anti-cancer strategies against leukaemia.

## Materials and Methods

### Cell culture

CCRF-CEM cells were isolated from peripheral blood of a child with ALL^96^. Multidrug-resistant, P-glycoprotein over-expressing CEM/ADR5000 cells were derived from CCRF-CEM cells by continuous treatment with doxorubicin up to a concentration of 5,000 ng/mL^97^. These cells were generously provided by Prof. Axel Sauerbrey (Department of Pediatrics, University of Jena, Jena, Germany). The cell lines were authenticated using Multiplex Cell Authentication (MCA) based on single nucleotide polymorphism profiling by Multiplexion GmbH (Heidelberg, Germany) as recently described^98^. Those cell lines have been in culture for over a time period of 14 years.

### RNA sequencing

CCRF-CEM cells and CEM/ADR5000 cells were used. Total RNA isolation was performed with InviTrap Spin Universal RNA Mini Kit (Stratec, Birkenfeld, Germany). Total RNA quality and quantity were evaluated using an Agilent Bioanalyser 2100 and Qubit Fluorometer (Life Technologies, Darmstadt, Germany). Poly A^+^ RNA was isolated, fractionated and double-stranded cDNA was synthesised using the TruSeq RNA sample prep v2 protocol (Illumina Inc., San Diego, CA). End-repaired, A-tailed and adaptor-ligated cDNA was PCR-amplified by 10 cycles. The library was sequenced in paired-end mode (2 × 100 bp) using 0.4 lane of an Illumina HiSeq 2000 flowcell. Gene expressions were quantified using the RPKM measure^25^,^99^. RPKM values for transcripts and the ratios of transcripts were taken into consideration to calculate the overall RPKM value for each gene. The deregulation of genes in CEM/ADR5000 cells was calculated by dividing overall RPKM values of genes in CEM/ADR5000 cells by those in CCRF-CEM cells. Chromosomal locations of deregulated genes were retrieved from UCSC Table Browser (https://genome.ucsc.edu/cgi-bin/hgTables). Genes involved in classical drug resistance mechanisms (ABC transporters, apoptosis, autophagy, CYP enzymes, DNA repair, ferroptosis, glutathione related enzymes, heat shock proteins, kinases, necroptosis, oxidative stress, receptors, topoisomerases and transcription factors) were retrieved from HUGO database^100^ and the literature to categorize the deregulated genes in resistance classes.

### Pathway and network analysis

Fold change in RNA expression of ±7 were applied for filtering and then subjected to Ingenuity Pathway Analysis (QIAGEN Redwood City, USA, www.qiagen.com/ingenuity) to identify specific networks and pathways in CEM/ADR5000 cells. Functional gene enrichment analyses were performed with the DAVID software^101^,^102^,^103^. SABiosciences PCR array (Qiagen, http://www.sabiosciences.com/ArrayList.php?pline=PCRArray) is another useful tool classifying genes in functional clusters. The genes residing in the following clusters were taken into account: cancer drug resistance, DNA damage signalling pathway, DNA repair, drug metabolism, drug metabolism phase I, drug metabolism phase II, drug transporters, heat shock proteins, oncogenes and tumour suppressors, oxidative stress, stem cell, transcription factors, Wnt signalling pathway.

### mFISH

CCRF-CEM and CEM/ADR5000 cells were cytogenetically prepared to obtain metaphase spreads and analysed using molecular cytogenetics as previously reported^104^. As probes, we used all 24 human whole chromosome paints in one experiment as previously reported^105^ as well as human multicolour banding^106^ probes as mentioned in Table 5. Additionally, partial chromosome paint (PCP), centromeric (CEP) or commercially available locus specific (LSI) probes were applied to verify or refine some of the multicolour FISH results, whole chromosome paint (WCP).

### aCGH

Whole genomic DNA was extracted from CCRF-CEM and CEM/ADR5000 cells with QIAmp DNA mini kit (QIAGEN GmbH, Hilden, Germany). aCGH was done as previously reported^107^.

### Western blotting

The protein expression levels of selected resistance genes, FOXO1 and NQO1 were evaluated in CCRF-CEM and CEM/ADR5000 cells to validate their deregulation found by RNA sequencing analysis. Protein isolation was performed using mPER protein extraction reagent (Invitrogen, Darmstadt, Germany) supplemented with protease inhibitor cocktail (Roche Diagnostics, Mannheim, Germany) according to the manufacturer’s recommendations. Concentrations of proteins were determined using NanoDrop1000 (PEQLAB, Erlangen, Germany). Overnight incubation at 4 °C with primary antibodies at a dilution of 1:1000 (anti- FOXO1-rabbit, anti-NQO1-mouse) or 1:2000 (anti-β-actin-rabbit) (New England Biolabs, Frankfurt, Germany) and then 2 h incubation at room temperature with HRP-linked secondary anti-rabbit or anti-mouse IgG antibody (1:2000 dilution) was performed. Detection was done with Luminata Classico HRP Western Blot substrate (Merck Millipore, Schwalbach, Germany).

## Additional Information

**How to cite this article**: Kadioglu, O. *et al*. Genomic and transcriptomic profiling of resistant CEM/ADR-5000 and sensitive CCRF-CEM leukaemia cells for unraveling the full complexity of multi-factorial multidrug resistance. *Sci. Rep.* **6**, 36754; doi: 10.1038/srep36754 (2016).

**Publisher’s note:** Springer Nature remains neutral with regard to jurisdictional claims in published maps and institutional affiliations.

## Supplementary Material

Supplementary Information

## Figures and Tables

**Table 1:** Most deregulated genes involved in classical resistance mechanisms in CEM/ADR5000 cells.

ABC transporter genes		Oxidative stress genes		Necroptosis genes	
Gene	Fold change	Gene	Fold change	Gene	Fold change
*ABCB1*	402.357	*PDLIM1*	270.419	*GLUL*	34.433
*ABCG2*	12.243	*HMOX1*	71.708		
*ABCA2*	10.496	*BNIP3*	10.407	**Receptor genes**	
		*CCDC88B*	−9.375	*NGFRAP1*	306.400
**Apoptosis genes**				*IL6R*	205.063
*TNFRSF10B*	44.890	**Heat shock genes**		*FZD7*	161.273
*HRK*	27.210	*DNAJC15*	−498.946	*PTGDR2*	−54.011
*BCL2L2*	24.963	*HSPH1*	−101.264		
*IGF1R*	14.600				
*TP73*	−121.42	**Kinase genes**		**CYP genes**	
		*IRAK3*	348.023	*CYP27B1*	13.229
**Transcription factor genes**		*PRKAR2A*	200.572		
*NKX3-1*	2848.955	*PRKCA*	70.938	**DNA repair genes**	
*KLF2*	417.710	*ITK*	−76.268	*NEIL2*	22.353
*SIX1*	363.432	*EPHA1*	−47.662		
*LIN28B*	−3367.714				
*ZNF501*	−186.938				

**Table 2:** Enriched biological functions and deregulated genes related to drug resistance as found by DAVID analysis.

P value	Fold enrichment	Gene ID	Fold change
**Leukocyte differentiation**			
7.4 × 10^−5^	3.8	*MMP9*	26.92
		*JAG2*	14.61
		*CEBPE*	11.25
		*CD8A*	−7.79
		*FLT3LG*	−10.19
		*BCL3*	−10.69
		*ITGA4*	−11.12
		*PTPN22*	−22.91
		*IKZF1*	−27.83
		*RAG1*	−48.56
		*CD28*	−50.42
		*CD79A*	−65.77
		*CD1D*	−353.72
**Regulation of exocytosis**			
2.3 × 10^−3^	6.3	*HMOX1*	71.71
		*PRKCA*	70.94
		*RAB3B*	11.86
		*TRPV6*	−7.13
		*PRAM1*	−12.88
**Membrane organisation**			
2.4 × 10^−3^	2.1	*EHD4*	579.74
		*LRP5*	31.8
		*AP1S3*	24.19
		*SYT7*	22.42
		*ARRB1*	19.94
		*STX11*	13.00
		*CEBPE*	11.25
		*MSR1*	10.45
		*BNIP3*	10.41
		*AGRN*	10.3
		*GATA2*	9.65
		*DNM3*	7.65
		*SYP*	7.17
		*RIN3*	−10.63
		*SH3KBP1*	−10.91
		*RAB34*	−11.87
		*APLP1*	−12.92
		*CD2*	−18.34
		*CD93*	−29.86
		*STAP1*	−43.77

**Table 3:** Deregulated genes residing at drug resistance related clusters.

Gene ID	Fold change	Functional cluster
ABCB1	402.36	Cancer drug resistance, drug metabolism, drug transporters
PDLIM1	270.42	Oxidative stress
FZD7	161.27	WNT signaling
CCND2	101.35	Stem cell, WNT signaling
FOXO1	80.08	Transcription factors
HMOX1	71.71	Oxidative stress
PRKCA	70.94	Oncogenes and tumour suppressors
LRP5	31.80	WNT signalling
CXADR	30.35	WNT signalling
GZMA	29.25	Drug metabolism, phase I
NEIL2	22.35	DNA repair
TST	21.91	Drug metabolism, phase II
DTX1	17.47	Stem cell
IGF1R	14.60	Cancer drug resistance
SLC2A3	13.64	Drug transporters
CYP27B1	13.23	Drug metabolism, phase I
PON2	12.25	Drug metabolism
ABCG2	12.24	Cancer drug resistance, drug transporters, stem cell
ABCA2	10.50	Drug transporters
BNIP3	10.41	Oxidative stress
BBC3	10.26	DNA damage
GATA2	9.65	Transcription factors
COL1A1	8.56	Stem cell
DNAJC15	−498.95	Heat shock
HSPH1	−101.26	Heat shock
AS3MT	−99.20	Drug metabolism, phase II
TCF7	−17.66	WNT signalling
CD44	−14.89	Stem cell
SLCO3A1	−12.43	Drug transporters
POU2AF1	−10.32	Transcription factors
SLC25A13	−9.88	Drug transporters
CD8A	−7.80	Stem cell

**Table 4:** Chromosomal aberrations and corresponding deregulated genes. Comparison between aCGH and RNA sequencing profiles. Significance levels were all below p<0.001.

Chr	Cyto-band	#Probes	Amp/Del	Annotated genes (up-regulated/down-regulated)
CEM-ADR5000				
chr1:4789122-6794876	p36.32–p36.31	109	−0.924274	*DNAJC11*
chr3:176180822-197845254	q26.32–q29	1336	−0.786136	*ABCC5, ABCF3, DNAJB11, DNAJC19*
chr4:150831733-179917923	q31.23–q34.3	1524	−0.866775	*NEIL3*
chr7:87067493-87250285	q21.12	13	2.392485	*ABCB1*
chr14:98604505-106705307	q32.2–q32.33	616	0.493302	*JAG2*
chr18:52985254-78010032	q21.2–q23	1297	−0.879675	*BCL2*
chr20:29842786-62904501	q11.21–q13.33	2217	0.500089	*MMP9*
chrX:2535073-57987522	p22.33–p11.21	3190	−0.856470	*SH3KBP1*
chrX:61931689-155097214	q11.1–q28	4813	−0.866241	*ABCD1*
CCRF-CEM				
chr5:172797353-180712263	q35.1–q35.3	480	−0.807537	*RAB24*
chr14:22636039-22964922	q11.2	30	−3.097243	*LRP10*
chr20:67778-26194459	p13–p11.1	1586	0.476161	*CD93*
chr20:29842786-62904501	q11.21–q13.33	2221	0.497165	*CEBPB, COL9A3, SLC9A8*

**Table 5:** FISH probes used for molecular cytogenetic characterisation of CCRF-CEM and CEM/ADR5000 cell lines.

Probe combinations for CCRF-CEM, P.21	Source
multiplex-FISH applying all 24 human wcp probes	homemade
LSI BCR/ABL	Vysis
MCB for chromosome 6	homemade
MCB for chromosome 7	homemade
MCB for chromosome 8	homemade
MCB for chromosome 9	homemade
TelVysion 5q	Vysis
WCP for chromosome 5	homemade
WCP for chromosome 14	homemade
TelVysion 9p	Vysis
CEP for chromosome 8	Vysis
CEP for chromosome 9	Vysis
TelVysion 14q	Vysis
WCP for chromosome 5	homemade
WCP for chromosome 14	homemade
**Probe combinations for CEM/ADR5000, P.47**	
multiplex-FISH applying all 24 human wcp probes	homemade
LSI BCR/ABL	Vysis
MCB for chromosome 3	homemade
MCB for chromosome 4	homemade
MCB for chromosome 5	homemade
MCB for chromosome 6	homemade
MCB for chromosome 7	homemade
MCB for chromosome 8	homemade
MCB for chromosome 9	homemade
MCB for chromosome 10	homemade
MCB for chromosome 13	homemade
MCB for chromosome 14	homemade
MCB for chromosome 16	homemade
MCB for chromosome 17	homemade
MCB for chromosome 18	homemade
MCB for chromosome 20	homemade
MCB for chromosome 21	homemade
MCB for chromosome 22	homemade
WCP for chromosome 6	homemade
WCP for chromosome 8	homemade
WCP for chromosome 20	homemade
WCP for chromosome 6	homemade
WCP for chromosome 14	homemade
TelVysion 3q	Vysis
CEP for chromosome 3	Vysis
CEP for chromosome 13/21	ZytoVision
TelVysion 4q	Vysis
CEP for chromosome 4	Vysis
TelVysion 5q	Vysis
WCP for chromosome 5	homemade
WCP for chromosome 14	homemade
TelVysion 6q	Vysis
CEP for chromosome 6	Vysis
CEP for chromosome 20	Vysis
TelVysion 6q	Vysis
CEP for chromosome 6	Vysis
CEP for chromosome 14/22	Kreatech
TelVysion 9p	Vysis
CEP for chromosome 8	Vysis
CEP for chromosome 9	Vysis
TelVysion 14q	Vysis
WCP for chromosome 5	Homemade
WCP for chromosome 14	homemade
Telvysion 21q	Vysis
CEP for chromosome 18	Vysis
CEP for chromosome 13/21	Vysis
Telvysion 22q	Vysis
CEP for chromosome 14/22	Vysis
CEP for chromosome 9	Vysis
NOR-specific probeSO	homemade
CEP for chromosome 18	Vysis
Midi 54 = probe for all acrocentric short arms	homemade
CEP for chromosome 18	Vysis
WCP for chromosome 17	homemade
PCP for chromosome 17q	homemade
WCP for chromosome 3	homemade
WCP for chromosome 14	homemade
WCP for chromosome 6	homemade
WCP for chromosome 20	homemade
Telvysion 21q	Vysis
CEP for chromosome 18	Vysis
WCP for chromosome 17	homemade

Abbreviations: CEP = centromeric probe; MCB = multicolour banding; PCP = partial chromosome paint; WCP = whole chromosome paint.
