# Supplementary material for: Genomic and transcriptomic profiling of resistant CEM/ADR-5000 and sensitive CCRF-CEM leukaemia cells for unravelling the full complexity of multi-factorial multidrug resistance
Source: Sci Rep. 2016 Nov 8;6:36754. doi: 10.1038/srep36754 (PMC5099876; doi:10.1038/srep36754)
Supplement: Supplementary Information [file srep36754-s1.pdf]

Genomic and transcriptomic profiling of resistant CEM/ADR-5000 and sensitive CCRF-CEM leukaemia cells for unravelling the full complexity of multi-factorial multidrug resistance

Onat Kadioglu[1], Jingming Cao[1], Nadezda Kosyakova[2], Kristin Mrasek[2], Thomas Liehr[2], Thomas Efferth[1]*

[1] Department of Pharmaceutical Biology, Institute of Pharmacy and Biochemistry, Johannes Gutenberg University, Mainz, Germany

[2] Jena University Hospital, Friedrich Schiller University, Institute of Human Genetics, Jena, Germany

* Corresponding author:

Prof. Dr. Thomas Efferth, Institute of Pharmacy and Biochemistry, Johannes Gutenberg University, Mainz, Staudinger Weg 5, 55128 Mainz, Germany

Tel: 49-6131-3925751, Fax: 49-6131-3923752, E-mail: efferth@uni-mainz.de

Running title: Gene expression profiling of CEM/ADR5000 and CCRF-CEM with RNA-sequencing, array-CGH and mFISH

Supplementary Table 1- Deregulated genes involved in classical resistance mechanisms in CEM/ADR5000 cells

Apoptosis (7/101)-% 7			
**Gene**	**Fold change**	**Gene**	**Fold change**
TNFRSF10B	44.890	*BNIP3*	10.407
HRK	27.210	*CD27*	-8.242
BCL2L2	24.963	*TP73*	-121.420
IGF1R	14.600		

Kinase (34/726)-%5			
**Gene**	**Fold change**	**Gene**	**Fold change**
IRAK3	348.023	*HCST*	-7.729
PRKAR2A	200.572	*AKIP1*	-8.981
PRKCA	70.938	*CERKL*	-9.345
INSR	47.644	*FLT3LG*	-10.193
CAMK2N1	46.176	*SH3KBP1*	-10.913
MAP3K8	28.771	*CAMK4*	-12.974
EFNB2	14.901	*CAMK1D*	-18.728
PRKAG2	12.321	*EPHA1*	-47.662
PRKAR2B	11.657	*ITK*	-76.268
TNIK	11.135	*HCST*	-7.729
PFKFB4	10.749	*AKIP1*	-8.981
MAGI1	10.733	*CERKL*	-9.345
CKB	9.505	*FLT3LG*	-10.193
SBK1	7.835	*SH3KBP1*	-10.913
PIK3AP1	7.331	*CAMK4*	-12.974
BMPR2	7.021	*CAMK1D*	-18.728
		EPHA1	-47.662
		ITK	-76.268

ABC transporters (3/48)-%6	
**Gene**	Fold change
ABCB1	402.357
ABCG2	12.243
ABCA2	10.496

CYP enzymes (1/47)-%2	
**Gene**	**Fold change**
CYP27B1	13.229

Heat shock (2/112)-%2	
**Gene**	**Fold change**
HSPH1	-101.264
DNAJC15	-498.946

Transcription factor (49/1469)-%3			
Gene	Fold change	Gene	Fold change
NKX3-1	2848.955	*HES6*	8.542
KLF2	417.710	*KLF7*	7.905
SIX1	363.432	*NFE2L3*	7.763
TCF4	106.481	*HOXD12*	7.375
FOXO1	80.077	*ZFP3*	-7.430
TSHZ3	54.152	*NR5A1*	-8.376
ARNTL2	37.317	*ZFAT*	-8.555
HEYL	37.253	*SOX5*	-8.619
ZBTB38	33.201	*ZNF141*	-9.182
HNF4G	32.905	*MYT1L*	-10.124
ZFHX3	30.452	*ZNF419*	-10.595
HES4	23.715	*ZNF34*	-11.789
ZNF620	21.764	*FOXH1*	-11.832
HMX3	17.600	*ZNF846*	-12.051
FOXF1	14.391	*ZNF844*	-12.224
HHEX	13.669	*ZNF544*	-12.705
IRX3	11.922	*BATF3*	-13.126
MYCL1	11.618	*TCF7*	-17.664
CEBPE	11.245	*IKZF1*	-27.829
ZNF697	9.705	*ZBTB47*	-42.886
GATA2	9.654	*ZNF585A*	-67.146
POU4F1	9.258	*AFF2*	-97.095
FOXA1	9.083	*ZNF501*	-186.938
HOXB7	8.704	*LIN28B*	-3367.714
NFIA	8.688		

DNA repair (2/225)-%1	
Gene	Fold change
NEIL2	22.353
MSH4	10.291

Necroptosis (1/77)-%0.5	
Gene	Fold change
GLUL	34.433

Oxidative stress (4/110)-%4	
Gene	Fold change
PDLIM1	270.419
HMOX1	71.708
BNIP3	10.407
CCDC88B	-9.375

Receptors (47/1522)-%3			
**Gene**	**Fold change**	**Gene**	**Fold change**
IRAK3	348.023	*GPR85*	8.806
NGFRAP1	306.400	*PTGER3*	8.709
IL6R	205.063	*PTPN13*	8.483
FZD7	161.273	*NPTXR*	8.039
PROCR	52.648	*IL21R*	7.870
LRP1	52.488	*SCARB2*	7.690
INSR	47.644	*RTN4RL2*	7.339
TNFRSF10B	44.890	*BMPR2*	7.021
ARNTL2	37.317	*TRPV6*	-7.126
LRP5	31.800	*F2R*	-7.677
CXADR	30.351	*IL2RB*	-7.698
BZRAP1	29.323	*NR5A1*	-8.376
TNFSF13B	26.488	*ITGA4*	-11.115
P2RY1	25.684	*AVPR1B*	-11.286
KISS1R	25.056	*MCHR1*	-16.955
ITGA3	18.175	*PTPN22*	-22.913
TNFRSF13C	16.375	*NCR3*	-23.970
GPR3	14.973	*FCGRT*	-26.428
IGF1R	14.600	*TRPM2*	-33.144
KDELR3	14.441	*TNFSF8*	-37.750
GPR135	11.154	*RTP4*	-44.650
MSR1	10.452	*EPHA1*	-47.662
IFNGR2	9.960	*PTGDR2*	-54.011
TNFSF9	9.343		

Supplementary Table 2-Detailed karyotypes of all subclones detected in this study for cell lines CCRF-CEM and CEM/ADR5000

Karyotypes of the 7 clones found in this study and a putative original karyotype of CCRF-CEM

**CCRF-CEM putative original karyotype:**<br>47,XX,der(5)t(5;14)(q35.33;q32.3),del(9)(p14.1),+20
**CCRF-CEM karyotype clone 1:**<br>47,XX,der(5)t(5;14)(q35.33;q32.3),t(8;9)(p12;p24),del(9)(p14.1),+20
**CCRF-CEM karyotype clone 2:**<br>46,X,-X,der(5)t(5;14)(q35.33;q32.3),del(9)(p14.1),+20
**CEM/ADR5000 clone 1:**<br>47,X,-X,t(3;10)(q11.2~12;p14~15),der(3)t(3;13)(q26.32;q22.3),del(4)(q31.32q34.3), der(5)t(18;5;14)(18qter→18q21.2::5p12→5q35.33::14q32.3→14qter),inv(7)(p21.1q21.1), t(8;9)(p12;p24),del(9)(p14.1),t(10;16)(q23.31;q22~23),del(14)(q32.3), der(18)t(7;18)(p21;q21.2),der(18)(21qter→21q22.1::18p11.22→18q12.1: :5p12→5pter),der(18)(21p?::21q22.3→21q22.1::18p11.22→18q12.1::5p12→5pter), +20,der(22)t(9;22)(q22.33;q13.33)
**CEM/ADR5000 clone 1a:**<br>47,X,-X,t(3;10)(q11.2~12;p14~15),der(3)t(3;13)(q26.32;q22.3),del(4)(q31.32q34.3), der(5)t(18;5;14)(18qter→18q21.2::5p12→5q35.33::14q32.3→14qter), t(6;14)(q26;q32.33),inv(7)(p21.1q21.1),t(8;9)(p12;p24),del(9)(p14.1),t(10;16) (q23.31;q22~23),del(14)(q32.3),der(18)t(7;18)(p21;q21.2),der(18)(21qter→21q22.1: :18p11.22→18q12.1::5p12→5pter),der(18)(21p?::21q22.3→21q22.1: :18p11.22→18q12.1::5p12→5pter),+20,der(22)t(9;22)(q22.33;q13.33)
**CEM/ADR5000 clone 1b:**<br>47,X,-X,der(3)t(3;10)(q11.2~12;p14~15),der(3)t(3;13)(q26.32;q22.3),del(4) (q31.32q34.3),der(5)t(18;5;14)(18qter→18q21.2::5p12→5q35.33::14q32.3→14qter), inv(7)(p21.1q21.1),t(8;9)(p12;p24),del(9)(p14.1),t(10;16)(q23.31;q22~23), der(10)t(20;3;10)(20pter→20p11.2::3q26.3→3q11.2~12::10p14~15→10qter), +del(14)(q32.3),der(18)t(7;18)(p21;q21.2),der(18)(21qter→21q22.1: :18p11.22→18q12.1::5p12→5pter),der(18)(21p?::21q22.3→21q22.1: :18p11.22→18q12.1::5p12→5pter),+,der(20)t(3;20)(q26.3;p11.2), der(22)t(9;22)(q22.33;q13.33)
**CEM/ADR5000 clone 1b1:**

47,X,-X,der(3)t(3;10)(q11.2~12;p14~15),der(3)t(3;13)(q26.32;q22.3),del(4)(q31.32q34.3),der(5)t(18;5;14)(18qter→18q21.2::5p12→5q35.33::14q32.3→14qter),inv(7)(p21.1q21.1),t(8;9)(p12;p24),del(9)(p14.1),t(10;16)(q23.31;q22~23),der(10)t(20;3;10)(20pter→20p11.2::3q26.3→3q11.2~12::10p14~15→10qter),+del(14)(q32.3),der(17)t(17;18;5)(17pter→17q22::18q11.22→18q12.1::5p12→5pter),der(18)t(7;18)(p21;q21.2),der(18)t(21;18;5)(21qter→21q22.12::18p11.22→18q11.22::17q22→17qter),+der(18)t(21;18;5)(21p?::21q22.3→21q22.12::18p11.22→18q12.1::5p12→5pter),der(20)t(3;20)(q26.3;p11.2),der(22)t(9;22)(q22.33;q13.33)
**CEM/ADR5000 clone 1c:**<br>46,X,-X,t(3;10)(q11.2~12;p14~15),der(3)t(3;13)(q26.32;q22.3),del(4)(q31.32q34.3),der(5)t(18;5;14)(18qter→18q21.2::5p12→5q35.33::14q32.3→14qter),t(6;14)(q26;q32.33),t(6;20;8)(q24;q11.2~1;q22.3~23),inv(7)(p21.1q21.1),t(8;9)(p12;p24),del(9)(p14.1),t(10;16)(q23.31;q22~23),del(14)(q32.3),der(18)t(7;18)(p21;q21.2),der(18)(21qter→21q22.1::18p11.22→18q12.1::5p12→5pter) or der(18)(21p?::21q22.3→21q22.1::18p11.22→18q12.1::5p12→5pter),+20,der(22)t(9;22)(q22.33;q13.33)

Supplementary Table 3- Deregulated resistance genes in CEM/ADR5000 cells and their chromosomal locus

Gene Symbol	Gene Name	Differential RNA expression in CEM/ADR5000 compared to CCRF-CEM	Chromosomal Gene Locus
ABCA1	ATP-binding cassette, sub-family A (ABC1), member 1	2.726	9q31.1
ABCA2	ATP-binding cassette, sub-family A (ABC1), member 2	10.496	9q34.3
ABCA5	ATP-binding cassette, sub-family A (ABC1), member 5	4.411	17q24.3
ABCB1	ATP-binding cassette, sub-family B (MDR/TAP), member 1	402.357	7q21.12
ABCB10	ATP-binding cassette, sub-family B (MDR/TAP), member 10	1.786	1q42.13
ABCB6	ATP-binding cassette, sub-family B (MDR/TAP), member 6	3.115	2q35
ABCB8	ATP-binding cassette, sub-family B (MDR/TAP), member 8	-1.79	7q36.1
ABCC2	ATP-binding cassette, sub-family C (CFTR/MRP), member 2	-1.643	10q24.2
ABCC5	ATP-binding cassette, sub-family C (CFTR/MRP), member 5	-2.641	3q27.1
ABCC6	ATP-binding cassette, sub-family C (CFTR/MRP), member 6	-2.219	16p13.11
ABCD1	ATP-binding cassette, sub-family D (ALD), member 1	-1.963	Xq28
ABCF1	ATP-binding cassette, sub-family F (GCN20), member 1	-1.572	6p21.33
ABCF3	ATP-binding cassette, sub-family F (GCN20), member 3	-1.612	3q27.1
ABCG2	ATP-binding cassette, sub-family G (WHITE), member 2	12.243	4q22.1
ABHD2	abhydrolase domain containing 2	1.974	15q26.1
ACSL1	acyl-CoA synthetase long-chain family member 1	-2.894	4q35.1
ACSL4	acyl-CoA synthetase long-chain family member 4	1.502	Xq23
ACSM3	acyl-CoA synthetase medium-chain family member 3	6.98	16p12.3
AIFM1	apoptosis-inducing factor, mitochondrion-associated, 1	-1.588	Xq26.1
AKT1	v-akt murine thymoma viral oncogene homolog 1	-1.765	14q32.33
ALCAM	activated leukocyte cell adhesion molecule	10.427	3q13.11
ALDH1A2	aldehyde dehydrogenase 1 family, member A2	1.711	15q21.3
ALDH1B1	aldehyde dehydrogenase 1 family, member B1	1.936	9p13.1
ALDH4A1	aldehyde dehydrogenase 4 family, member A1	-2.251	1p36.13
ALDH5A1	aldehyde dehydrogenase 5 family, member A1	-2.343	6p22.3
ANAPC2	anaphase promoting complex subunit 2	1.693	9q34.3
ANXA1	annexin A1	-2.005	9q21.13
ANXA4	annexin A4	1.591	2p13.3
APAF1	apoptotic peptidase activating factor 1	1.648	12q23.1
APC	adenomatous polyposis coli	2.726	5q22.2
API5	apoptosis inhibitor 5	-1.924	11p12
AS3MT	arsenite methyltransferase	-99.2	10q24.32
ATF2	activating transcription factor 2	-1.558	2q31.1
ATF3	activating transcription factor 3	4.099	1q32.3
ATF4	activating transcription factor 4	-2.082	22q13.1
ATG12	autophagy related 12	-1.575	5q22.3
ATG4B	autophagy related 4B, cysteine peptidase	-1.79	2q37.3

ATG4C	autophagy related 4C, cysteine peptidase	-1.933	1p31.3
ATG4D	autophagy related 4D, cysteine peptidase	-2.115	19p13.2
ATM	ATM serine/threonine kinase	1.695	11q22.3
ATP6V0C	ATPase, H+ transporting, lysosomal 16kDa, V0 subunit c	-1.61	16p13.3
ATRIP	ATR interacting protein	1.742	3p21.31
ATRX	alpha thalassemia/mental retardation syndrome X-linked	-1.84	Xq21.1
AVEN	apoptosis, caspase activation inhibitor	1.561	15q14
BAD	BCL2-associated agonist of cell death	-2.278	11q13.1
BAG3	BCL2-associated athanogene 3	1.709	10q26.11
BAX	BCL2-associated X protein	-1.546	19q13.33
BBC3	BCL2 binding component 3	10.26	19q13.32
BCAT1	branched chain amino-acid transaminase 1, cytosolic	-2.59	12p12.1
BCL10	B-cell CLL/lymphoma 10	3.064	1p22.3
BCL2	B-cell CLL/lymphoma 2	-3.178	18q21.33
BCL2L11	BCL2-like 11 (apoptosis facilitator)	1.581	2q13
BCL2L2	BCL2-like 2	24.963	14q11.2
BCL3	B-cell CLL/lymphoma 3	-10.697	19q13.32
BCLAF1	BCL2-associated transcription factor 1	1.654	6q23.3
BID	BH3 interacting domain death agonist	-2.483	22q11.21
BIRC2	baculoviral IAP repeat containing 2	-2.862	11q22.2
BIRC3	baculoviral IAP repeat containing 3	-2.567	11q22.2
BNIP3	BCL2/adenovirus E1B 19kDa interacting protein 3	10.407	10q26.3
BNIP3L	BCL2/adenovirus E1B 19kDa interacting protein 3-like	1.835	8p21.2
BOK	BCL2-related ovarian killer	1.533	2q37.3
BRCA1	breast cancer 1, early onset	-2.051	17q21.31
BRCA2	breast cancer 2, early onset	2.678	13q13.1
BTG2	BTG family, member 2	-2.016	1q32.1
BTK	Bruton agammaglobulinemia tyrosine kinase	-1.949	Xq22.1
CA9	carbonic anhydrase IX	5.208	9p13.3
CAPN3	calpain 3, (p94)	-1.995	15q15.1
CAPN5	calpain 5	1.62	11q13.5
CAPNS1	calpain, small subunit 1	3.45	19q13.12
CARD11	caspase recruitment domain family, member 11	1.53	7p22.2
CASP3	caspase 3, apoptosis-related cysteine peptidase	2.678	4q35.1
CASP4	caspase 4, apoptosis-related cysteine peptidase	-2.048	11q22.3
CASP6	caspase 6, apoptosis-related cysteine peptidase	-2.21	4q25
CASP7	caspase 7, apoptosis-related cysteine peptidase	2.524	10q25.3
CASP8	caspase 8, apoptosis-related cysteine peptidase	-1.58	2q33.1
CASP9	caspase 9, apoptosis-related cysteine peptidase	3.235	1p36.21
CCL5	chemokine (C-C motif) ligand 5	-4.397	17q12
CCNC	cyclin C	2.206	6q16.2

CCND2	cyclin D2	101.346	12p13.32
CCND3	cyclin D3	-1.728	6p21.1
CD2	CD2 molecule	-18.338	1p13.1
CD27	CD27 molecule	-8.242	12p13.31
CD28	CD28 molecule	-50.423	2q33.2
CD38	CD38 molecule	-2.588	4p15.32
CD5	CD5 molecule	-84.505	11q12.2
CD70	CD70 molecule	-2.257	19p13.3
CDC34	cell division cycle 34	-1.72	19p13.3
CDK6	cyclin-dependent kinase 6	-2.088	7q21.2
CDK9	cyclin-dependent kinase 9	-1.741	9q34.11
CDKN2C	cyclin-dependent kinase inhibitor 2C (p18, inhibits CDK4)	-3.81	1p32.3
CDKN3	cyclin-dependent kinase inhibitor 3	1.646	14q22.2
CEBPB	CCAAT/enhancer binding protein (C/EBP), beta	-1.944	20q13.13
CEL	carboxyl ester lipase	2.165	9q34.13
CFD	complement factor D (adipsin)	14.875	19p13.3
CIB1	calcium and integrin binding 1 (calmyrin)	-1.949	15q26.1
CKM	creatine kinase, muscle	-1.814	19q13.32
COL15A1	collagen, type XV, alpha 1	-4.036	9q22.33
COMT	catechol-O-methyltransferase	-3.689	22q11.21
CREB1	cAMP responsive element binding protein 1	-2.7	2q33.3
CREM	cAMP responsive element modulator	1.518	10p11.21
CTSB	cathepsin B	-1.724	8p23.1
CTSD	cathepsin D	-2.357	11p15.5
CUL2	cullin 2	1.524	10p11.21
CXCR4	chemokine (C-X-C motif) receptor 4	-2.316	2q22.1
CYLD	cylindromatosis (turban tumor syndrome)	-2.262	16q12.1
CYP27B1	cytochrome P450, family 27, subfamily B, polypeptide 1	13.229	12q14.1
CYP2E1	cytochrome P450, family 2, subfamily E, polypeptide 1	1.931	10q26.3
CYP4F2	cytochrome P450, family 4, subfamily F, polypeptide 2	-1.968	19p13.12
DACH1	dachshund family transcription factor 1	-2.337	13q21.33
DBP	D site of albumin promoter (albumin D-box) binding protein	-3.847	19q13.33
DDIT3	DNA-damage-inducible transcript 3	-2.594	12q13.3
DDOST	dolichyl-diphosphooligosaccharide--protein glycosyltransferase subunit (non-catalytic)	-1.859	1p36.12
DEDD	death effector domain containing	1.935	1q23.3
DHCR24	24-dehydrocholesterol reductase	2.774	1p32.3
DLL4	delta-like 4 (Drosophila)	4.37	15q15.1
DMC1	DNA meiotic recombinase 1	-3.993	22q13.1
DNMT3B	DNA (cytosine-5-)-methyltransferase 3 beta	1.985	20q11.21
DPYD	dihydropyrimidine dehydrogenase	-1.594	1p21.3
DR1	down-regulator of transcription 1, TBP-binding (negative cofactor 2)	-1.831	1p22.1

DUSP1	dual specificity phosphatase 1	-1.621	5q35.1
EGR1	early growth response 1	2.468	5q31.2
EPHX1	epoxide hydrolase 1, microsomal (xenobiotic)	-1.708	1q42.12
EPX	eosinophil peroxidase	-3	17q22
ETS1	v-ets avian erythroblastosis virus E26 oncogene homolog 1	-2.192	11q24.3
ETS2	v-ets avian erythroblastosis virus E26 oncogene homolog 2	5.326	21q22.2
EZH2	enhancer of zeste 2 polycomb repressive complex 2 subunit	-1.629	7q36.1
F2R	coagulation factor II (thrombin) receptor	-7.677	5q13.3
FAIM	Fas apoptotic inhibitory molecule	1.503	3q22.3
FBXW7	F-box and WD repeat domain containing 7, E3 ubiquitin protein ligase	-2.565	4q31.3
FEN1	flap structure-specific endonuclease 1	-1.861	11q12.2
FHL1	four and a half LIM domains 1	-6.764	Xq26.3
FIGF	c-fos induced growth factor (vascular endothelial growth factor D)	3.069	Xp22.2
FLT4	fms-related tyrosine kinase 4	-1.906	5q35.3
FMO5	flavin containing monooxygenase 5	2.893	1q21.1
FOXO1	forkhead box O1	80.077	13q14.11
FZD7	frizzled class receptor 7	161.273	2q33.1
GAA	glucosidase, alpha; acid	1.513	17q25.3
GATA2	GATA binding protein 2	9.654	3q21.3
GATA3	GATA binding protein 3	-3.051	10p14
GLUL	glutamate-ammonia ligase	34.433	1q25.3
GNMT	glycine N-methyltransferase	1.542	6p21.1
GRB2	growth factor receptor-bound protein 2	-1.775	17q25.1
GSK3A	glycogen synthase kinase 3 alpha	-2.431	19q13.2
GSTA4	glutathione S-transferase alpha 4	1.829	6p12.2
GSTK1	glutathione S-transferase kappa 1	1.828	7q34
GSTM4	glutathione S-transferase mu 4	2.864	1p13.3
GSTO1	glutathione S-transferase omega 1	2.009	10q25.1
GSTP1	glutathione S-transferase pi 1	-4.003	11q13.2
GSTZ1	glutathione S-transferase zeta 1	-1.582	14q24.3
GZMA	granzyme A (granzyme 1, cytotoxic T-lymphocyte-associated serine esterase 3)	29.245	5q11.2
HDAC1	histone deacetylase 1	-1.876	1p35.2
HDAC2	histone deacetylase 2	-1.572	6q21
HDAC6	histone deacetylase 6	1.597	Xp11.23
HIC1	hypermethylated in cancer 1	1.79	17p13.3
HIP1	huntingtin interacting protein 1	1.622	7q11.23
HIPK2	homeodomain interacting protein kinase 2	-2.038	7q34
HMOX1	heme oxygenase (decycling) 1	71.708	22q12.3
HNF1A	HNF1 homeobox A	2.321	12q24.31
HRK	harakiri, BCL2 interacting protein	27.21	12q24.22
HSPA1B	heat shock 70kDa protein 1A	2.859	6p21.33

HSPA4L	heat shock 70kDa protein 4-like	4.24	4q28.1
HSPB1	heat shock 27kDa protein 1	-1.975	7q11.23
HSPBAP1	HSPB (heat shock 27kDa) associated protein 1	-1.936	3q21.1
HSPH1	heat shock 105kDa/110kDa protein 1	-101.264	13q12.3
IER3	immediate early response 3	-2.279	6p21.33
IFI16	interferon, gamma-inducible protein 16	-6.691	1q23.1
IGF1R	insulin-like growth factor 1 receptor	14.6	15q26.3
IGF2R	insulin-like growth factor 2 receptor	-1.985	6q25.3
IKBKG	inhibitor of kappa light polypeptide gene enhancer in B-cells, kinase gamma	-2.149	Xq28
IL1A	interleukin 1, alpha	2.071	2q14.1
IRF1	interferon regulatory factor 1	-1.818	5q31.1
IRGM	immunity-related GTPase family, M	-1.775	5q33.1
ITGA2	integrin, alpha 2 (CD49B, alpha 2 subunit of VLA-2 receptor)	2.45	5q11.2
ITGA4	integrin, alpha 4 (antigen CD49D, alpha 4 subunit of VLA-4 receptor)	-11.115	2q31.3
ITGA6	integrin, alpha 6	-11.07	2q31.1
ITGB1	integrin, beta 1 (fibronectin receptor, beta polypeptide, antigen CD29 includes MDF2, MSK12)	-1.686	10p11.22
ITPR2	inositol 1,4,5-trisphosphate receptor, type 2	4.259	12p11.23
JAG1	jagged 1	5.049	20p12.2
JAG2	jagged 2	14.611	14q32.33
JUN	jun proto-oncogene	2.184	1p32.1
JUND	jun D proto-oncogene	1.562	19p13.11
LAMP1	lysosomal-associated membrane protein 1	2.074	13q34
LCK	LCK proto-oncogene, Src family tyrosine kinase	-4.965	1p35.2
LDHA	lactate dehydrogenase A	-1.84	11p15.1
LGALS1	lectin, galactoside-binding, soluble, 1	3.187	22q13.1
LIG4	ligase IV, DNA, ATP-dependent	4.553	13q33.3
LIN28B	lin-28 homolog B (C. elegans)	-3367.714	6q16.3
LTB	lymphotoxin beta (TNF superfamily, member 3)	-18.812	6p21.33
MALT1	mucosa associated lymphoid tissue lymphoma translocation gene 1	2.316	18q21.32
MAOA	monoamine oxidase A	2.068	Xp11.3
MAP1LC3B	microtubule-associated protein 1 light chain 3 beta	-2.747	16q24.2
MAP3K5	mitogen-activated protein kinase kinase kinase 5	4.247	6q23.3
MCM3	minichromosome maintenance complex component 3	-1.506	6p12.2
MCM5	minichromosome maintenance complex component 5	-1.504	22q12.3
MDM2	MDM2 proto-oncogene, E3 ubiquitin protein ligase	1.918	12q15
MGAT1	mannosyl (alpha-1,3-)-glycoprotein beta-1,2-N-acetylglucosaminyltransferase	-1.697	5q35.3
MGAT2	mannosyl (alpha-1,6-)-glycoprotein beta-1,2-N-acetylglucosaminyltransferase	-1.84	14q21.3
MGST2	microsomal glutathione S-transferase 2	3.159	4q31.1
MKI67	marker of proliferation Ki-67	1.506	10q26.2
MMP9	matrix metallopeptidase 9 (gelatinase B, 92kDa	26.923	20q13.12

	gelatinase, 92kDa type IV collagenase)		
MOAP1	modulator of apoptosis 1	-1.789	14q32.12
MPG	N-methylpurine-DNA glycosylase	-2.132	16p13.3
MRE11A	MRE11 meiotic recombination 11 homolog A (S. cerevisiae)	-1.699	11q21
MSH5	mutS homolog 5	-1.529	6p21.33
MSH6	mutS homolog 6	-3.093	2p16.3
MSRA	methionine sulfoxide reductase A	-1.551	8p23.1
MTL5	metallothionein-like 5, testis-specific (tesmin)	-1.835	11q13.3
MTOR	mechanistic target of rapamycin (serine/threonine kinase)	1.703	1p36.22
MUTYH	mutY homolog	-2.724	1p34.1
MX1	MX dynamin-like GTPase 1	2.309	21q22.3
MYC	v-myc avian myelocytomatosis viral oncogene homolog	-1.972	8q24.21
MYO18A	myosin XVIIIA	3.343	17q11.2
NAIP	NLR family, apoptosis inhibitory protein	-2.438	5q13.2
NCOA7	nuclear receptor coactivator 7	-2.138	6q22.31
NDUFA13	NADH dehydrogenase (ubiquinone) 1 alpha subcomplex, 13	-2.392	19p13.11
NEIL2	nei endonuclease VIII-like 2 (E. coli)	22.353	8p23.1
NEIL3	nei endonuclease VIII-like 3 (E. coli)	-2.756	4q34.3
NEXN	nexilin (F actin binding protein)	-3.004	1p31.1
NFAT5	nuclear factor of activated T-cells 5, tonicity-responsive	5.453	16q22.1
NFATC1	nuclear factor of activated T-cells, cytoplasmic, calcineurin-dependent 1	-1.902	18q23
NFATC3	nuclear factor of activated T-cells, cytoplasmic, calcineurin-dependent 3	-1.737	16q22.1
NFKB2	nuclear factor of kappa light polypeptide gene enhancer in B-cells 2 (p49/p100)	-1.982	10q24.32
NFKBIB	nuclear factor of kappa light polypeptide gene enhancer in B-cells inhibitor, beta	-1.946	19q13.2
NGFRAP1	nerve growth factor receptor (TNFRSF16) associated protein 1	306.4	Xq22.2
NLRP1	NLR family, pyrin domain containing 1	-1.793	17p13.2
NME3	NME/NM23 nucleoside diphosphate kinase 3	-2.859	16p13.3
NOD1	nucleotide-binding oligomerization domain containing 1	-1.869	7p14.3
NOL3	nucleolar protein 3 (apoptosis repressor with CARD domain)	1.833	16q22.1
NOTCH3	notch 3	3.108	19p13.12
NPC1	Niemann-Pick disease, type C1	1.627	18q11.2
NQO1	NAD(P)H dehydrogenase, quinone 1	-3.57	16q22.1
NQO2	NAD(P)H dehydrogenase, quinone 2	1.539	6p25.2
NR3C1	nuclear receptor subfamily 3, group C, member 1 (glucocorticoid receptor)	-6.338	5q31.3
PARP3	poly (ADP-ribose) polymerase family, member 3	-1.917	3p21.2
PAX6	paired box 6	-3.366	11p13
PCNA	proliferating cell nuclear antigen	-1.515	20p12.3
PDLIM1	PDZ and LIM domain 1	270.419	10q23.33
PEA15	phosphoprotein enriched in astrocytes 15	1.557	1q23.2
PIDD1	p53-induced death domain protein 1	-2.059	11p15.5

PIK3C2A	phosphatidylinositol-4-phosphate 3-kinase, catalytic subunit type 2 alpha	2.176	11p15.1
PIK3C3	phosphatidylinositol 3-kinase, catalytic subunit type 3	2.679	18q12.3
PIK3CA	phosphatidylinositol-4,5-bisphosphate 3-kinase, catalytic subunit alpha	-2.622	3q26.32
PLAT	plasminogen activator, tissue	-1.943	8p11.21
PLAUR	plasminogen activator, urokinase receptor	2.11	19q13.31
PLEKHF1	pleckstrin homology domain containing, family F (with FYVE domain) member 1	-2.298	19q12
PLK1	polo-like kinase 1	-1.997	16p12.2
PMAIP1	phorbol-12-myristate-13-acetate-induced protein 1	-5.229	18q21.32
PMS1	PMS1 postmeiotic segregation increased 1 (S. cerevisiae)	-1.534	2q32.2
PMS2	PMS2 postmeiotic segregation increased 2 (S. cerevisiae)	2.263	7p22.1
PNKP	polynucleotide kinase 3'-phosphatase	-1.917	19q13.33
POMGNT1	protein O-linked mannose N-acetylglucosaminyltransferase 1 (beta 1,2-)	1.55	1p34.1
POU2AF1	POU class 2 associating factor 1	-10.324	11q23.1
POU4F1	POU class 4 homeobox 1	9.258	13q31.1
PPARA	peroxisome proliferator-activated receptor alpha	-2.381	22q13.31
PPID	peptidylprolyl isomerase D	-1.966	4q32.1
PRDX3	peroxiredoxin 3	1.642	10q26.11
PRKAB2	protein kinase, AMP-activated, beta 2 non-catalytic subunit	1.511	1q21.1
PRKCA	protein kinase C, alpha	70.938	17q24.2
PRKCB	protein kinase C, beta	-1.562	16p12.2
PRKCD	protein kinase C, delta	-2.74	3p21.1
PRKCE	protein kinase C, epsilon	2.148	2p21
PTCH1	patched 1	-4.44	9q22.32
PVR	poliovirus receptor	4.459	19q13.31
RAB24	RAB24, member RAS oncogene family	1.52	5q35.3
RAD21	RAD21 homolog (S. pombe)	-1.625	8q24.11
RAD23A	RAD23 homolog A (S. cerevisiae)	-1.801	19p13.13
RAD51	RAD51 recombinase	-2.024	15q15.1
RAD51B	RAD51 paralog B	-1.856	14q24.1
RAD52	RAD52 homolog (S. cerevisiae)	-1.575	12p13.33
RAD54L	RAD54-like (S. cerevisiae)	-1.746	1p34.1
RAD9A	RAD9 homolog A (S. pombe)	-3.771	11q13.2
RAF1	Raf-1 proto-oncogene, serine/threonine kinase	-1.588	3p25.2
RB1	retinoblastoma 1	-1.572	13q14.2
REL	v-rel avian reticuloendotheliosis viral oncogene homolog	1.581	2p16.1
RELA	v-rel avian reticuloendotheliosis viral oncogene homolog A	-2.044	11q13.1
REV1	REV1, polymerase (DNA directed)	1.813	2q11.2
RIPK3	receptor-interacting serine-threonine kinase 3	2.2	14q12
RND1	Rho family GTPase 1	7.053	12q13.12
RTN4	reticulon 4	-1.501	2p16.1

RUNX1	runt-related transcription factor 1	1.573	21q22.12
RXRA	retinoid X receptor, alpha	4.373	9q34.2
S100A4	S100 calcium binding protein A4	1.909	1q21.3
SAT1	spermidine/spermine N1-acetyltransferase 1	-3.516	Xp22.11
SAV1	salvador family WW domain containing protein 1	1.816	14q22.1
SEMA4D	sema domain, immunoglobulin domain (Ig), transmembrane domain (TM) and short cytoplasmic domain, (semaphorin) 4D	-2.331	9q22.2
SIK1	salt-inducible kinase 1	2.335	21q22.3
SKP2	S-phase kinase-associated protein 2, E3 ubiquitin protein ligase	1.649	5p13.2
SLC15A2	solute carrier family 15 (oligopeptide transporter), member 2	3.475	3q13.33
SLC19A2	solute carrier family 19 (thiamine transporter), member 2	1.888	1q24.2
SLC25A13	solute carrier family 25 (aspartate/glutamate carrier), member 13	-9.878	7q21.3
SLC25A4	solute carrier family 25 (mitochondrial carrier; adenine nucleotide translocator), member 4	1.622	4q35.1
SLC2A3	solute carrier family 2 (facilitated glucose transporter), member 3	13.642	12p13.31
SLC38A5	solute carrier family 38, member 5	1.765	Xp11.23
SLC3A1	solute carrier family 3 (amino acid transporter heavy chain), member 1	2.835	2p21
SLC3A2	solute carrier family 3 (amino acid transporter heavy chain), member 2	-1.71	11q12.3
SLC5A3	solute carrier family 5 (sodium/myo-inositol cotransporter), member 3	-1.769	21q22.11
SLC7A11	solute carrier family 7 (anionic amino acid transporter light chain, xc- system), member 11	2.652	4q28.3
SLC7A8	solute carrier family 7 (amino acid transporter light chain, L system), member 8	2.045	14q11.2
SLCO3A1	solute carrier organic anion transporter family, member 3A1	-12.429	15q26.1
SMAD1	SMAD family member 1	-1.538	4q31.21
SMAD5	SMAD family member 5	3.15	5q31.1
SMC1A	structural maintenance of chromosomes 1A	-1.631	Xp11.22
SMO	smoothened, frizzled class receptor	-1.63	7q32.1
SMUG1	single-strand-selective monofunctional uracil-DNA glycosylase 1	1.951	12q13.13
SOCS1	suppressor of cytokine signaling 1	3.083	16p13.13
SOCS2	suppressor of cytokine signaling 2	5.723	12q22
SOCS3	suppressor of cytokine signaling 3	22.182	17q25.3
SSTR3	somatostatin receptor 3	-2.975	22q13.1
STAT5A	signal transducer and activator of transcription 5A	2.648	17q21.2
STAT5B	signal transducer and activator of transcription 5B	-1.863	17q21.2
STK17A	serine/threonine kinase 17a	-1.53	7p13
STK17B	serine/threonine kinase 17b	-3.16	2q32.3
SULT1A2	sulfotransferase family, cytosolic, 1A, phenol-preferring, member 2	-1.5	16p11.2
TAZ	tafazzin	-1.554	Xq28
TBK1	TANK-binding kinase 1	1.811	12q14.2
TBP	TATA box binding protein	1.903	6q27
TCF4	transcription factor 4	106.481	18q21.2
TERT	telomerase reverse transcriptase	-1.901	5p15.33

TFDP2	transcription factor Dp-2 (E2F dimerization partner 2)	-1.891	3q23
TGFB1	transforming growth factor, beta 1	-1.625	19q13.2
TIMP1	TIMP metallopeptidase inhibitor 1	-2.377	Xp11.3
TMEM74	transmembrane protein 74	3.709	8q23.1
TNF	tumor necrosis factor	-2.298	6p21.33
TNFRSF10B	tumor necrosis factor receptor superfamily, member 10b	44.89	8p21.3
TNFRSF10D	tumor necrosis factor receptor superfamily, member 10d, decoy with truncated death domain	1.87	8p21.3
TNFRSF14	tumor necrosis factor receptor superfamily, member 14	-6.178	1p36.32
TNFSF10	tumor necrosis factor (ligand) superfamily, member 10	-2.24	3q26.31
TNFSF8	tumor necrosis factor (ligand) superfamily, member 8	-37.75	9q33.1
TOP1	topoisomerase (DNA) I	-1.695	20q12
TOP2A	topoisomerase (DNA) II alpha 170kDa	-6.831	17q21.2
TOP2B	topoisomerase (DNA) II beta 180kDa	-2.342	3p24.2
TOP3B	topoisomerase (DNA) III beta	-2.202	22q11.22
TP53	tumor protein p53	-1.6	17p13.1
TP53BP1	tumor protein p53 binding protein 1	1.952	15q15.3
TP53BP2	tumor protein p53 binding protein 2	1.751	1q41
TPMT	thiopurine S-methyltransferase	2.804	6p22.3
TPO	thyroid peroxidase	5.471	2p25.3
TRAF2	TNF receptor-associated factor 2	-1.95	9q34.3
TRAF3	TNF receptor-associated factor 3	-1.969	14q32.32
TRAF5	TNF receptor-associated factor 5	2.166	1q32.3
TSC1	tuberous sclerosis 1	1.78	9q34.13
TSC22D3	TSC22 domain family, member 3	5.439	Xq22.3
TST	thiosulfate sulfurtransferase (rhodanese)	21.909	22q12.3
TUBB6	tubulin, beta 6 class V	3.409	18p11.21
TXN	thioredoxin	2.247	9q31.3
TXNL4B	thioredoxin-like 4B	-1.741	16q22.2
TXNRD1	thioredoxin reductase 1	-1.513	12q23.3
UBXN2A	UBX domain protein 2A	-1.527	2p23.3
UCP2	uncoupling protein 2 (mitochondrial, proton carrier)	-1.84	11q13.4
ULK1	unc-51 like autophagy activating kinase 1	-1.501	12q24.33
UTP11L	UTP11-like, U3 small nucleolar ribonucleoprotein (yeast)	-1.542	1p34.3
UVRAG	UV radiation resistance associated	-1.621	11q13.5
XAB2	XPA binding protein 2	-2.837	19p13.2
XIAP	X-linked inhibitor of apoptosis	-1.909	Xq25
XPA	xeroderma pigmentosum, complementation group A	1.738	9q22.33
XRCC3	X-ray repair complementing defective repair in Chinese hamster cells 3	-1.587	14q32.33
ZEB1	zinc finger E-box binding homeobox 1	2.483	10p11.22
ZHX2	zinc fingers and homeoboxes 2	-2.98	8q24.13

ZNF148	zinc finger protein 148	-1.913	3q21.2

Supplementary Table 4- Deregulated resistance genes and corresponding chromosomal aberrations in CEM/ADR5000 cells

Gene Symbol	Gene Name	Differential RNA expression in CEM/ADR5000 compared to CCRF-CEM	Chromosomal Gene Locus	Chromosomal Aberration
ABCB1	ATP-binding cassette, sub-family B (MDR/TAP), member 1	402.357	7q21.12	Amplification (2.392485)
ABCC5	ATP-binding cassette, sub-family C (CFTR/MRP), member 5	-2.641	3q27.1	Deletion (-0.786136)
ABCD1	ATP-binding cassette, sub-family D (ALD), member 1	-1.963	Xq28	Deletion (-0.866241)
ABCF3	ATP-binding cassette, sub-family F (GCN20), member 3	-1.612	3q27.1	Deletion (-0.786136)
ACSL4	acyl-CoA synthetase long-chain family member 4	1.502	Xq23	Deletion (-0.866241)
ACSM3	acyl-CoA synthetase medium-chain family member 3	6.98	16p12.3	Deletion (-0.762611)
AIFM1	apoptosis-inducing factor, mitochondrion-associated, 1	-1.588	Xq26.1	Deletion (-0.866241)
AKT1	v-akt murine thymoma viral oncogene homolog 1	-1.765	14q32.33	Amplification (0.493302)
ATRX	alpha thalassemia/mental retardation syndrome X-linked	-1.84	Xq21.1	Deletion (-0.866241)
BAD	BCL2-associated agonist of cell death	-2.278	11q13.1	Amplification (0.715071)
BCL2	B-cell CLL/lymphoma 2	-3.178	18q21.33	Deletion (-0.879675)
BTK	Bruton agammaglobulinemia tyrosine kinase	-1.949	Xq22.1	Deletion (-0.866241)
CARD11	caspase recruitment domain family, member 11	1.53	7p22.2	Amplification (0.613966)
CD27	CD27 molecule	-8.242	12p13.31	Amplification (0.58453)
CEBPB	CCAAT/enhancer binding protein (C/EBP), beta	-1.944	20q13.13	Amplification (0.500089)
DNMT3B	DNA (cytosine-5-)-methyltransferase 3 beta	1.985	20q11.21	Amplification (0.500089)
ETS2	v-ets avian erythroblastosis virus E26 oncogene homolog 2	5.326	21q22.2	Amplification (0.833532)
FBXW7	F-box and WD repeat domain containing 7, E3 ubiquitin protein ligase	-2.565	4q31.3	Deletion (-0.866775)
FIGF	c-fos induced growth factor (vascular endothelial growth factor D)	3.069	Xp22.2	Deletion (-0.85647)
FLT4	fms-related tyrosine kinase 4	-1.906	5q35.3	Deletion (-0.766882)
GAA	glucosidase, alpha; acid	1.513	17q25.3	Amplification (0.507684)
JAG1	jagged 1	5.049	20p12.2	Amplification (0.492198)
JAG2	jagged 2	14.611	14q32.33	Amplification (0.493302)
MALT1	mucosa associated lymphoid tissue lymphoma translocation gene 1	2.316	18q21.32	Deletion (-0.879675)
MAOA	monoamine oxidase A	2.068	Xp11.3	Deletion (-0.85647)
MMP9	matrix metallopeptidase 9 (gelatinase B, 92kDa gelatinase, 92kDa type IV collagenase)	26.923	20q13.12	Amplification (0.500089)
MX1	MX dynamin-like GTPase 1	2.309	21q22.3	Amplification (0.833532)
NEIL3	nei endonuclease VIII-like 3 (E. coli)	-2.756	4q34.3	Deletion (-0.866775)
NGFRAP1	nerve growth factor receptor (TNFRSF16) associated protein 1	306.4	Xq22.2	Deletion (-0.866241)
NPC1	Niemann-Pick disease, type C1	1.627	18q11.2	Amplification (0.516186)
PCNA	proliferating cell nuclear antigen	-1.515	20p12.3	Amplification (0.492198)
PIK3CA	phosphatidylinositol-4,5-bisphosphate 3-kinase, catalytic subunit alpha	-2.622	3q26.32	Deletion (-0.786136)
PLK1	polo-like kinase 1	-1.997	16p12.2	Deletion (-0.762611)

PMAIP1	phorbol-12-myristate-13-acetate-induced protein 1	-5.229	18q21.32	Deletion (-0.879675)
PMS2	PMS2 postmeiotic segregation increased 2 (S. cerevisiae)	2.263	7p22.1	Amplification (0.613966)
PPID	peptidylprolyl isomerase D	-1.966	4q32.1	Deletion (-0.866775)
RAB24	RAB24, member RAS oncogene family	1.52	5q35.3	Deletion (-0.766882)
SAT1	spermidine/spermine N1-acetyltransferase 1	-3.516	Xp22.11	Deletion (-0.85647)
SIK1	salt-inducible kinase 1	2.335	21q22.3	Amplification (0.833532)
SKP2	S-phase kinase-associated protein 2, E3 ubiquitin protein ligase	1.649	5p13.2	Amplification (0.414735)
SLC2A3	solute carrier family 2 (facilitated glucose transporter), member 3	13.642	12p13.31	Amplification (0.58453)
SLC38A5	solute carrier family 38, member 5	1.765	Xp11.23	Deletion (-0.85647)
SMC1A	structural maintenance of chromosomes 1A	-1.631	Xp11.22	Deletion (-0.85647)
TAZ	tafazzin	-1.554	Xq28	Deletion (-0.866241)
TCF4	transcription factor 4	106.481	18q21.2	Deletion (-0.879675)
TERT	telomerase reverse transcriptase	-1.901	5p15.33	Amplification (0.414735)
TIMP1	TIMP metallopeptidase inhibitor 1	-2.377	Xp11.3	Deletion (-0.85647)
TNFRSF10B	tumor necrosis factor receptor superfamily, member 10b	44.89	8p21.3	Amplification (0.69472)
TOP1	topoisomerase (DNA) I	-1.695	20q12	Amplification (0.500089)
TRAF3	TNF receptor-associated factor 3	-1.969	14q32.32	Amplification (0.493302)
TSC22D3	TSC22 domain family, member 3	5.439	Xq22.3	Deletion (-0.866241)
UBXN2A	UBX domain protein 2A	-1.527	2p23.3	Deletion (-0.825474)
XIAP	X-linked inhibitor of apoptosis	-1.909	Xq25	Deletion (-0.866241)

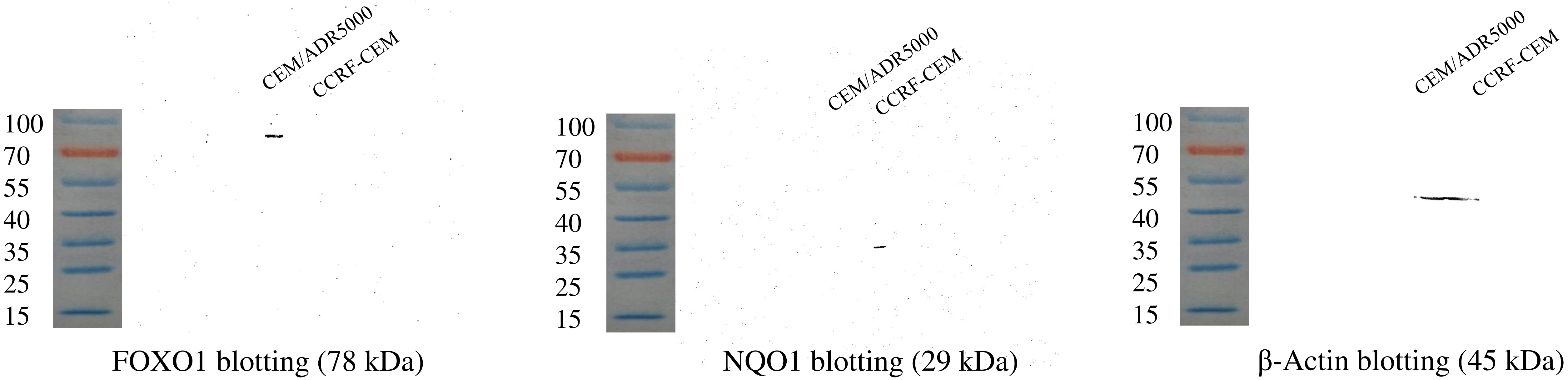

Supplementary Figure 1- Protein expression of FOXO1 and NQO1 in CEM/ADR5000 and CCRF-CEM cells as determined by western blotting (full blots are displayed)
